# Integration of spatial and single-cell transcriptomics localizes epithelial cell–immune cross-talk in kidney injury

**DOI:** 10.1172/jci.insight.147703

**Published:** 2021-06-22

**Authors:** Ricardo Melo Ferreira, Angela R. Sabo, Seth Winfree, Kimberly S. Collins, Danielle Janosevic, Connor J. Gulbronson, Ying-Hua Cheng, Lauren Casbon, Daria Barwinska, Michael J. Ferkowicz, Xiaoling Xuei, Chi Zhang, Kenneth W. Dunn, Katherine J. Kelly, Timothy A. Sutton, Takashi Hato, Pierre C. Dagher, Tarek M. El-Achkar, Michael T. Eadon

**Affiliations:** 1 Department of Medicine and; 2 Department of Medical and Molecular Genetics, Indiana University School of Medicine, Indianapolis, Indiana, USA.

**Keywords:** Nephrology, Expression profiling, Mouse models

## Abstract

Single-cell sequencing studies have characterized the transcriptomic signature of cell types within the kidney. However, the spatial distribution of acute kidney injury (AKI) is regional and affects cells heterogeneously. We first optimized coordination of spatial transcriptomics and single-nuclear sequencing data sets, mapping 30 dominant cell types to a human nephrectomy. The predicted cell-type spots corresponded with the underlying histopathology. To study the implications of AKI on transcript expression, we then characterized the spatial transcriptomic signature of 2 murine AKI models: ischemia/reperfusion injury (IRI) and cecal ligation puncture (CLP). Localized regions of reduced overall expression were associated with injury pathways. Using single-cell sequencing, we deconvoluted the signature of each spatial transcriptomic spot, identifying patterns of colocalization between immune and epithelial cells. Neutrophils infiltrated the renal medulla in the ischemia model. *Atf3* was identified as a chemotactic factor in S3 proximal tubules. In the CLP model, infiltrating macrophages dominated the outer cortical signature, and *Mdk* was identified as a corresponding chemotactic factor. The regional distribution of these immune cells was validated with multiplexed CO-Detection by indEXing (CODEX) immunofluorescence. Spatial transcriptomic sequencing complemented single-cell sequencing by uncovering mechanisms driving immune cell infiltration and detection of relevant cell subpopulations.

## Introduction

Acute kidney injury (AKI) is a devastating disease with a negative effect on morbidity and mortality. Developing therapeutic targets to treat AKI requires a better grasp of its molecular pathogenesis. Despite important advances in understanding this disease, the pathogenesis of AKI at the cellular and molecular levels remains incompletely understood. This is partially due to the diverse renal milieu of heterogeneous cell types (epithelial, endothelial, fibroblast, vascular smooth muscle, resident immune, and infiltrating immune cells) that interact with each other within a cosmos of unique microenvironments. Furthermore, AKI differentially affects the kidney’s diverse array of cells (1). Recently, single-cell and single-nuclear sequencing have proved major breakthroughs in the creation of a molecular atlas of the kidney (2–5) by defining the transcriptomic signatures of specific cells within the kidney. However, spatial anchoring is essential to understanding the relationship between cells and structures within specific renal microenvironments.

Single-cell and single-nuclear sequencing afford indirect spatial localization. In contrast, spatial transcriptomic platforms enable measurement of whole-transcriptome mRNA expression of thousands of genes superimposed upon histological information from the same tissue section. Gene expression profiles are mapped back to their original location, enabling a direct link between histology and gene expression (6). Integration of the single-cell sequencing with spatial transcriptomics improves power and enables in situ visualization of signatures with mapping of a greater number of cell types than spatial transcriptomics alone (7). Together, the 2 orthogonal data sets allow determination of where immune cells reside in proximity to other cells in disease states and how regional injury influences the signature of epithelial cells.

In the present study, we utilized single-nuclear and single-cell sequencing data sets to map cell types back to spatial transcriptomic anchoring landmarks (spots) overlaid upon the human and murine kidney. We first optimized the methodology in the human kidney. We then examined differentially expressed genes (DEGs) and pathways in murine ischemia/reperfusion injury (IRI) and cecal ligation puncture (CLP) models of AKI. We used the transcriptomic signatures derived from the 10× Genomics Visium spatial gene expression platform to colocalize immune cells with epithelial cells, defining the distribution of immune cell transcript expression in these models. Key chemotactic factors expressed in epithelial cells were identified that contributed to immune cell infiltration and cross-talk in each injury model. Immune cell–type distributions were validated with spatially resolved multiplexed CO-Detection by indEXing (CODEX) immunofluorescence. Together, the complementary single-cell and spatial transcriptomic data sets are synergistic, enabling the development of a transcriptomic atlas of the kidney in health and disease, with direct correlation to histopathology. These tools will enhance the diagnostic capability of renal pathologists interpreting manifestations of AKI in kidney tissue.

## Results

### Unsupervised mapping and cell-type identification within the spatial transcriptomic map of the human kidney.

We sought to map transcriptomic signatures directly upon a histological section from a human reference nephrectomy stained with H&E. The tissue was obtained from a 59-year-old woman with minimal glomerular obsolescence and interstitial fibrosis (each affecting less than 10% of the glomeruli or renal parenchyma, respectively). No arteriolar hyalinosis was observed. An OCT compound–embedded tissue section underwent H&E staining and microscopy followed by permeabilization, RNA isolation, and sequencing with spatially localized barcodes to map back the transcriptomic signature directly upon the histological image.

**Figure 1:**
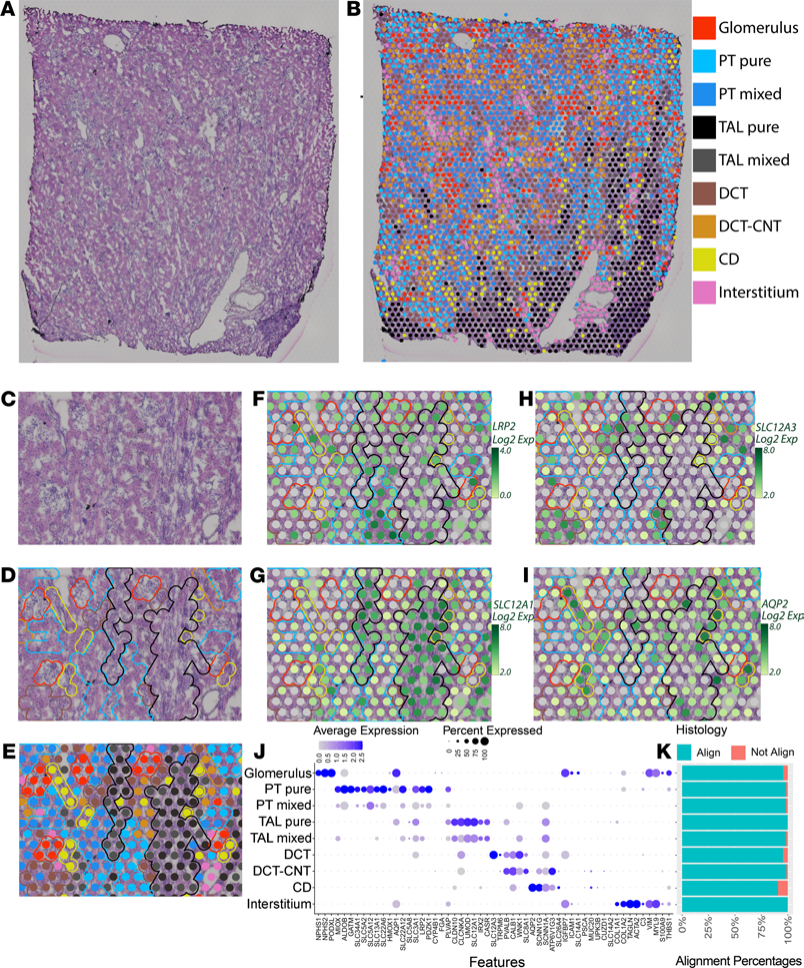
Spatial transcriptomics in a human nephrectomy sample. (**A**) H&E staining of the human reference nephrectomy. (**B**) The 9 unsupervised spatial transcriptomic (ST) clusters are overlaid upon the nephrectomy. Glomeruli can be seen scattered across the cortex in red. A medullary ray is seen in the right lower quadrant of the sample. Midsized vessels are often overlaid by pink interstitial cluster ST spots. Pure clusters are defined as those located mainly over the associated structure; mixed clusters often overlap with neighboring structures. (**C**) A high-magnification image showing histological structures in a reference nephrectomy. (**D**) Histological structures are highlighted in the nephrectomy. (**E**) Unsupervised clusters overlaid upon the nephrectomy. (**F**–**I**) Expression levels of *LRP2*, *SLC12A1*, *SLC12A3*, and *AQP2* in the spots over the high magnification region with histological features highlighted. (**J**) Expression of markers used to classify unsupervised clusters. (**K**) In 5 random fields covering 40% of all spots, the histology underlying each spot was assessed and the percentage of concordance is provided. All clusters held greater than 90% concordance with their corresponding histology. *n =* 1 human nephrectomy. PT, proximal tubule; S1, S2, S3, segments of PT; TAL, thick ascending limb; DCT, distal convoluted tubule; CNT, connecting tubule; CD, collecting duct. Each spot is 55 μm in diameter.

The result of the spatial transcriptomic mapping was a set of “spots” (55 μm in diameter), each with its own expression signature. These spots were overlaid upon the histological image of the kidney based on the localization barcodes (Figure 1, A and B) and clustered in an unsupervised fashion according to the gene expression of each spot. The identity of these clusters was established and named according to known kidney regions and cell types using differentially expressed marker genes and the underlying histology. Nine unsupervised clusters were generated by Space Ranger (Figure 1B), with expression signatures aligning with known marker genes of glomeruli and various tubular subsegments. These clusters were overlaid upon histological features, including glomeruli, tubules, vascular structures, and medullary rays, which were readily observed. On average, 10,270 counts were measured and 3205 genes were detected per spot. The average number of unique genes detected was 17,506 per cluster, with at least 16,000 detected in every cluster.

Spots assigned to a given cluster consistently localized over the expected underlying histology. An example of cluster mapping and identification is provided in Figure 1, C–I. A subset of marker genes is provided in Figure 1J and the full set is in Supplemental Table 1; supplemental material available online with this article; https://doi.org/10.1172/jci.insight.147703DS1 A close-up of the H&E section is provided without (Figure 1C) and with (Figure 1, D and E) unsupervised clusters overlaid. Glomeruli, small vessels, tubules, and a portion of the medullary rays can be visualized. Transcriptomic spots associated with glomerular genes (red) clearly overlie histologically identified glomeruli. The interstitial cluster, which includes expression of fibroblast, vascular smooth muscle cell (VSMC), and endothelial cell marker genes, is seen located over vessels (pink). Although tubular morphology in OCT-embedded sections is imperfect, distinctions can still be observed. The spatial transcriptomic–defined proximal tubule (PT) clusters overlie eosinophilic tubules without visible lumens. This histological pattern corresponded with megalin (*LRP2*) expression (Figure 1F). Spots assigned to thick ascending limb (TAL) clusters were congregated over medullary rays and correlated with expression of the loop diuretic sensitive sodium-potassium 2-chloride transporter (*SLC12A1*, Figure 1G). Distal convoluted tubules (DCTs) and connecting tubules (CNTs) were characterized by eosinophilic tubules with open lumens. DCTs had high corresponding expression of the thiazide-sensitive sodium-chloride transporter (*SLC12A3*, Figure 1H). Collecting ducts (CDs) were identified histologically with their characteristic nuclei in cuboidal cells and reduced eosinophilia. These tubules were overlaid by spots with high aquaporin-2 (*AQP2*) expression (Figure 1I).

Since each spatial transcriptomic spot was 55 μm in diameter, roughly the size of a tubular cross-section, the spots can overlie multiple cell types. Two PT and 2 TAL clusters were obtained in the unsupervised mapping. One PT and 1 TAL cluster each had stronger enrichment of known marker genes and were labeled “pure.” The clusters with less enrichment of marker genes or signatures with elements of 2 or more cell types were labeled “mixed.” The mixed TAL signature was seen on the periphery of the medullary rays, and the pure cluster was more centrally located (Figure 1B).

As stated, spots were classified into clusters based on the known gene expression markers of renal cell types (Figure 1J and Supplemental Table 1). The spots classified based on their gene expression signature mapped to expected histological/renal structures consistently. We assessed the consistence of the assigned transcriptomic cluster to the underlying histology in 5 randomly selected fields (Figure 1K). The overall correlation between assigned clusters and histological structures was 97.6%. All clusters mapped to a corresponding histological structure at greater than 95% accuracy except the CD (90.7%), which had the fewest overall spots in the specimen. The most frequent reason for discordance was the lack of significant tissue underlying a spot (e.g., a spot at the edge or within a large vessel).

### Mapping of single-nuclear sequencing clusters to human kidney tissue.

**Figure 2:**
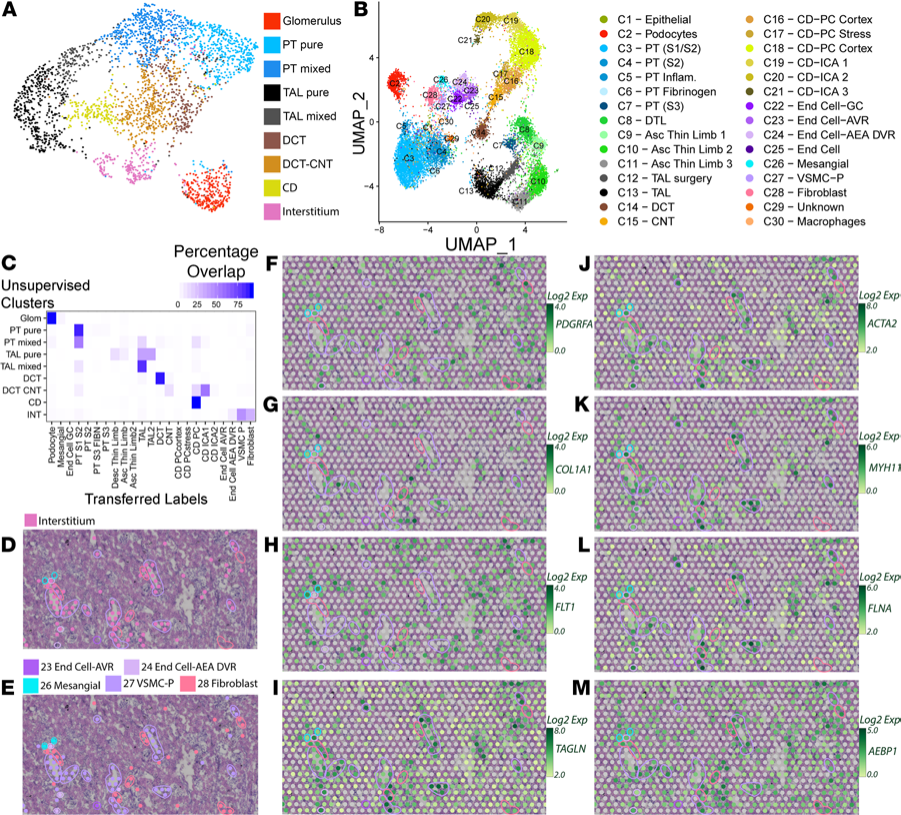
Transfer of single-nuclei RNA sequencing clusters to the human spatial transcriptomic sample. (**A**) UMAP projection of spatial transcriptomic (ST) data with 9 unsupervised clusters defined by Space Ranger. Spots assigned to the pure PT or thick ascending limb clusters were more frequently located over their corresponding histology; mixed clusters often overlapped neighboring structures. (**B**) A UMAP projection of the single-nucleus RNA-Seq data (GSE121862) depicts the 30 kidney cell clusters obtained from Pagoda. (**C**) The percentage of ST spots overlapping between unsupervised cluster spot identities and supervised cluster identities defined by single-nuclei expression signatures. Strong correlation is seen between expected clusters. Each row of the table adds to 100%. (**D**) A high-magnification image of the H&E-stained human reference nephrectomy with unsupervised interstitium cluster spots overlaid. Histological structures are highlighted. (**E**) A high-magnification image of the H&E-stained reference nephrectomy with mapped single-nucleus clusters associated with interstitium and histological structures highlighted. (**F**–**M**) Feature plots depict the expression levels of interstitial cell-type markers, such as *PDGFRA*, *COL1A1*, *FLT1*, *TAGLN*, *ACTA2*, *MYH11*, *FLNA*, and *AEBP1,* in the high-magnification region. Histological features highlighted. PT, proximal tubule; S1, S2, S3, segments of PT; TAL, thick ascending limb; DCT, distal convoluted tubule; CNT, connecting tubule; CD, collecting duct; DTL, descending thin limb; Asc, ascending; PC, principal cells; IC, intercalated cells; End, endothelial; GC, glomerular capsule; AVR, ascending vasa recta; AEA, afferent and efferent arterioles; DVR, descending vasa recta; VSMC-P, vascular smooth cells and pericytes. Each spot is 55 μm in diameter.

A uniform manifold approximation and projection (UMAP) of the unsupervised clusters obtained through Space Ranger is provided in Figure 2A. To improve the specificity of spot mapping and identify less frequent cell types that may contribute to the signature of a spot, we reclustered a publicly available single-nuclear RNA sequencing (snRNA-Seq) data set from human kidney samples (Figure 2B and ref. 2). The full list of DEG markers of each single-cell RNA sequencing (scRNA-Seq) cluster is in Supplemental Table 2. Thirty cell-type clusters were identified. In a process similar to single-cell cluster integration (8), transfer scores were assigned to spatial transcriptomic spots for each of the 30 snRNA-Seq clusters based on anchoring of common neighbors. Each spot was then labeled according to the snRNA-Seq cluster with the highest transfer score (Figure 2C). The percentage of spots overlapping between the unsupervised spatial transcriptomic clusters and those relabeled according to the snRNA-Seq transfer scores was assessed. A strong correlation was found between the unsupervised spatial transcriptomic classified spots and the spots redefined with the expected snRNA-Seq transfer labels.

Several cell types that were not represented in the unsupervised spatial transcriptomic clusters had spots assigned to them after snRNA-Seq relabeling. Since each spot covered multiple cells, the cell type was assigned to the most dominant cell type in that 55 μm spot. For example, multiple cell types contribute to the expression signature of the interstitium (9). The inclusion of snRNA-Seq data resolved the spots localizing in the interstitium to a more specific set of cell types (Figure 2, D–M). This data set allowed discrimination of spots assigned to fibroblasts, VSMCs, and some endothelium subtypes. Feature plots of known markers for the interstitium are shown in Figure 2, F–M.

### Cell-type deconvolution.

**Figure 3:**
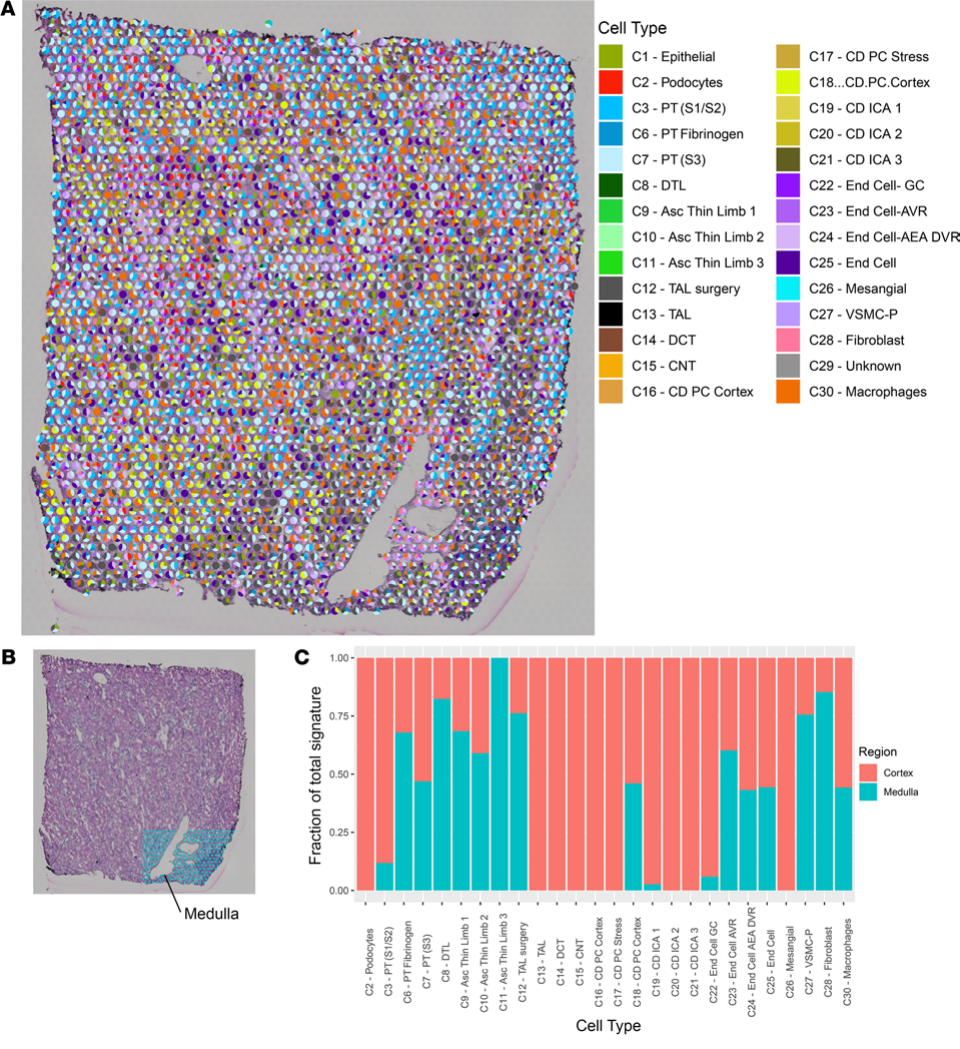
Neural network analysis of human kidney. (**A**) Each pie chart represents the contribution of the cell types from the single-nuclei reference data set to the transcriptomic signature of each spot in the human nephrectomy. Only cell types contributing to at least 10% of the spot signature are displayed. (**B**) An inset depicts the medullary region highlighted in blue (manually annotated). The remaining nephrectomy was considered cortex. (**C**) Fraction of total signature of each cell type present in the cortex and medulla, normalized to the total number of spots in the cortex and medulla. Each bar is calculated as follows: the proportion of expression arising from each spot was summed for each cell type in both the cortex and medulla. Summed expression was first normalized for (divided by) the number of spots in the cortex and medulla and then expressed as a ratio of expression arising from the cortex or medulla for each cell type. PT, proximal tubule; S1, S2, S3, segments of PT; TAL, thick ascending limb; DCT, distal convoluted tubule; CNT, connecting tubule; CD, collecting duct; DTL, descending thin limb; Asc, ascending; PC, principal cells; IC, intercalated cells; End, endothelial; GC, glomerular capsule; AVR, ascending vasa recta; AEA afferent and efferent arterioles; DVR, descending vasa recta; VSMC-P, vascular smooth cells and pericytes. Each spot is 55 μm in diameter.

Since each spot may overlie multiple cell types, we sought to determine the contribution of each snRNA-Seq cluster to the expression signature of each spot in a neural network analysis (Figure 3). As expected, the podocyte, mesangial cell, and endothelial cell clusters contributed the greatest proportion of signature to spots overlying glomeruli. A portion of medulla was included in the right lower portion of the nephrectomy sample. We quantitated the proportion of signature arising from each snRNA-Seq cluster in the medulla as compared with the remaining sample. Expression of genes defining the descending thin limb, ascending thin limb, and thick ascending loop of Henle were enriched in the medulla. As expected, no expression of podocyte signature and minimal contribution from S1 and S2 PTs were observed in the medulla. Two cortical CD clusters had been identified in the original snRNA-Seq data set (C16 and C18); however, in the spatial transcriptomic data set, the signature of C18 was found in both the cortex and medulla, while C16 mapped exclusively to the cortex.

### Spatial transcriptomic cell-type localization in the murine kidney.

**Figure 4:**
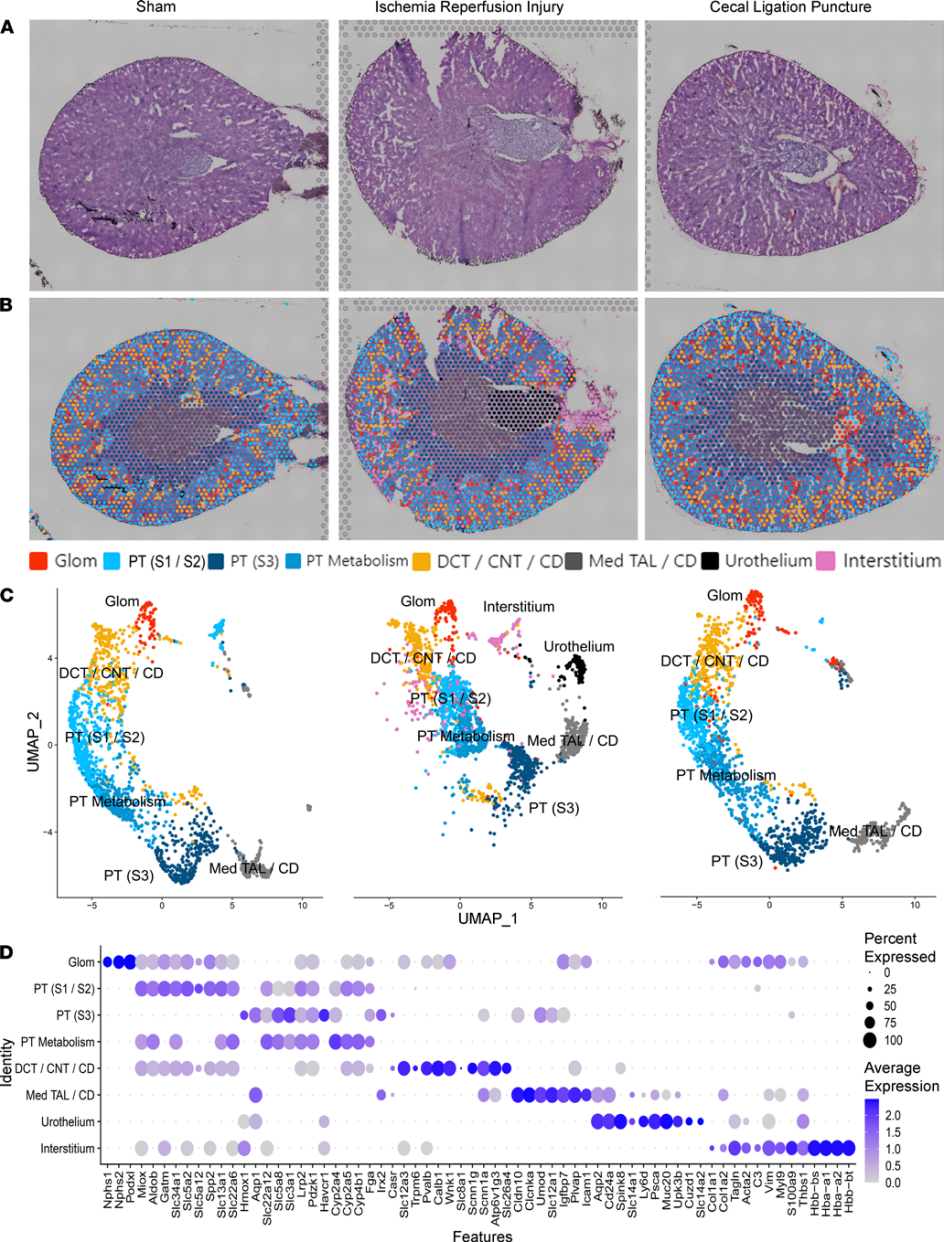
Spatial transcriptomics in murine kidney injury models. (**A**) H&E-stained sections of the 3 murine models: sham, ischemia/reperfusion injury (IRI), and cecal ligation puncture (CLP), respectively. (**B**) Spatial transcriptomic spots are overlaid upon each murine kidney derived from unbiased clustering. (**C**) A UMAP of the spatial clusters after the data was merged, split by tissue of origin (sham on left, IRI in middle, and CLP on right). (**D**) Expression of markers used to classify the spatial transcriptomic clusters. *n =* 1 murine sample per model. PT, proximal tubule; S1, S2, S3, segments of PT; Med, medullary; TAL, thick ascending limb; DCT, distal convoluted tubule; CNT, connecting tubule; CD, collecting; Glom, glomerulus. Each spot is 55 μm in diameter.

AKI does not affect all regions of the kidney uniformly. The etiology of injury may differentially affect the spatial distribution of gene expression, especially in the early course of disease. Therefore, we applied spatial transcriptomics on 2 common murine models of AKI (IRI and CLP), and the mice were euthanized at 6 hours. The unsupervised spatial transcriptomic cluster mapping is shown in Figure 4 for the sham, IRI, and CLP models. In matched cross-sections of the 3 models, minimal histopathological injury was observed with H&E staining at the 6-hour time point (Figure 4A). At low-power magnification, the IRI tissue area was larger (25.5 mm^2^) than the sham (16.9 mm^2^) and CLP (15.9 mm^2^) sections. Figure 4B overlays each section with the clusters obtained from Space Ranger. Two clusters were only present in the IRI model: the interstitium and urothelium clusters, the latter possibly because of the size of the papilla in the section. The 3 models were merged and normalized to allow comparisons between models (Figure 4C). A full set of DEGs between unsupervised spatial transcriptomic clusters is in Supplemental Table 3. Murine cells and tubules are smaller than their human counterparts, so each 55 μm spot is labeled by its dominant contributor. Fewer unsupervised clusters were identified in the mouse than the human. For example, the DCT, CNT, and CD of the distal nephron all clustered together. Similarly, the PT S3 segment cluster localized to the outer stripe and contained gene expression from the neighboring TAL. A subset of marker genes used to identify the clusters is depicted in Figure 4D. A specific comparison of the PT cluster DEGs is in Supplemental Figure 1.

### Regional expression differences and pathway enrichment in injury models.

The expression of 2 common markers of injury, kidney injury molecule-1 (*Havcr1*) and neutrophil gelatinase-associated lipocalin (*Lcn2*), were plotted in each model (Supplemental Figure 2). The distribution of *Havcr1* expression was localized to the outer stripe in the IRI model and not upregulated in the CLP model. *Lcn2* expression was diffusely upregulated in both models.

**Figure 5:**
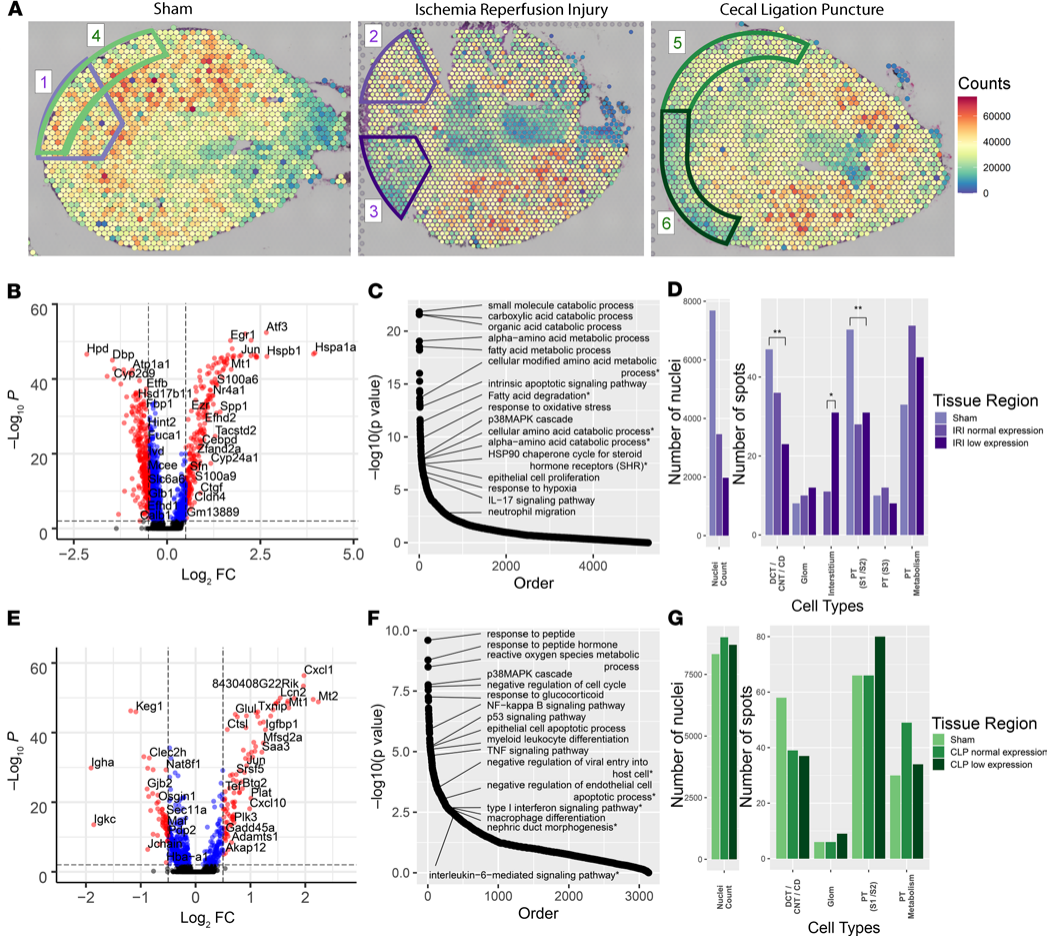
Regional expression in murine kidney injury models. (**A**) Total expression in read counts was summed for each spatial transcriptomic (ST) spot and the total expression level was overlaid upon each of the 3 murine model sections: sham (left), ischemia/reperfusion injury (IRI, middle), and cecal ligation puncture (CLP, right). Regions of interest and comparator regions are highlighted. In the sham, areas are selected to serve as reference to IRI (1, outlined in purple) and to CLP (4, outlined in green). In the IRI section, region 2 corresponds to the relatively “preserved” overall expression region and region 3 corresponds to a region of low relative expression. In the CLP section, analogous regions of preserved expression (5) and low expression (6) were selected. The regions were defined with similar areas within each comparison. (**B**) Volcano plot comparing the low-expression region in the IRI to the equivalent region in the sham. Despite the overall reduced expression of the IRI region, many individual genes were upregulated in IRI (right). (**C**) Pathways enriched for the differentially expressed genes (DEGs) between the low-expression region in IRI when compared with the sham. (**D**) Bar plots showing the number of nuclei and number of spots of each cluster in the 3 purple comparison regions. The asterisks indicate the significance level (*P < 0.1, **P < 0.001 as calculated by Fisher’s exact test. (**E**) Volcano plot comparing the low-expression region in the CLP to the equivalent region in the sham with upregulated genes in CLP on the right. (**F**) Pathways enriched for the DEGs between the low--expression region in CLP when compared with sham. (**G**) Bar plots showing the number of nuclei and number of spots of each cluster in the 3 green comparison regions. Each spot is 55 μm in diameter.

In the cortex of both models, we observed regional differences in the overall expression level of each spot as measured in total read counts across all genes (Figure 5A). We selected circumscribed regions of the IRI and CLP models that had areas of reduced expression when compared with the sham, despite minimal histopathological evidence of injury. These regions of “low” expression in IRI and CLP were compared with size-matched regions with relatively “preserved” expression. The low-expression regions were also compared with identically sized regions in the sham.

The DEGs, across all spots regardless of cluster identity, were uncovered in the IRI low-expression region and the equivalent sham region (Figure 5B). Despite the overall reduction in total read counts in IRI, a number of genes were significantly upregulated in the low-expression region. The enriched pathways based on the DEGs are presented in Figure 5C with a subset of pathways annotated. Several enriched pathways were identified related to metabolism of amino acids and fatty acids, possibly related to a metabolic shift associated with hypoxia (10–14). Other pathways suggest prominent injury response mechanisms, such as apoptosis, oxidative stress, and the p38 MAPK cascade (15–17). IL-17 signaling and enrichment of neutrophil migration indicate a potential inflammatory response of neutrophils in the IRI model (18–20). The full pathway list is in Supplemental Table 4 and DEGs are in Supplemental Table 5.

In a separate comparison, the IRI low- and preserved-expression regions were compared (Supplemental Tables 4 and 5). Similar DEGs and pathways were identified, albeit at lower levels of significance. The IRI preserved-expression region fell along the spectrum of expression changes between the IRI low-expression region and the sham region. We assessed the total nuclei count and spot distribution in each of these selected regions (Figure 5D). The sham region had approximately twice the nuclei of the IRI normal region and 4-fold more nuclei than the IRI low region. This finding is consistent with the overall changes in total regional expression. We speculate that this relative loss of nuclei in the IRI regions was due to interstitial edema because widespread apoptosis is not expected 6 hours after IRI. Furthermore, the change in the distribution of spot identity is also supportive, wherein the IRI model had an increased number of spots assigned to the interstitial cluster compared with the sham region, which had no spots mapping to the interstitial cluster. A reduced number of epithelial cells in the spots of the injured tissue may account for this shift in distribution. Indeed, fewer spots were found to map to tubular clusters (DCT/CNT/CD and PT [S1/S2]) in the IRI model.

An equivalent analysis was performed for the CLP model (Supplemental Tables 4 and 5). The DEGs and enriched pathways between the CLP low and sham regions were consistent with known changes in the CLP model, including p53 signaling, cell cycle arrest, apoptosis, TNF signaling, and macrophage differentiation (Figure 5, E and F). The preserved CLP region had muted expression differences and pathway enrichment, again falling along the differential expression spectrum between the CLP low and sham comparison. In contrast to the IRI model, differences in the nuclei count and the spot cluster identity distribution were not found (Figure 5G). Together, the expression patterns, nuclei count, and similarity in spot identity distribution between regions in the CLP model suggest a very different injury pattern than IRI. As compared with IRI, CLP injury may lead to less space occupying interstitial edema.

### Mapping of single-cell sequencing clusters to murine kidney tissue.

**Figure 6:**
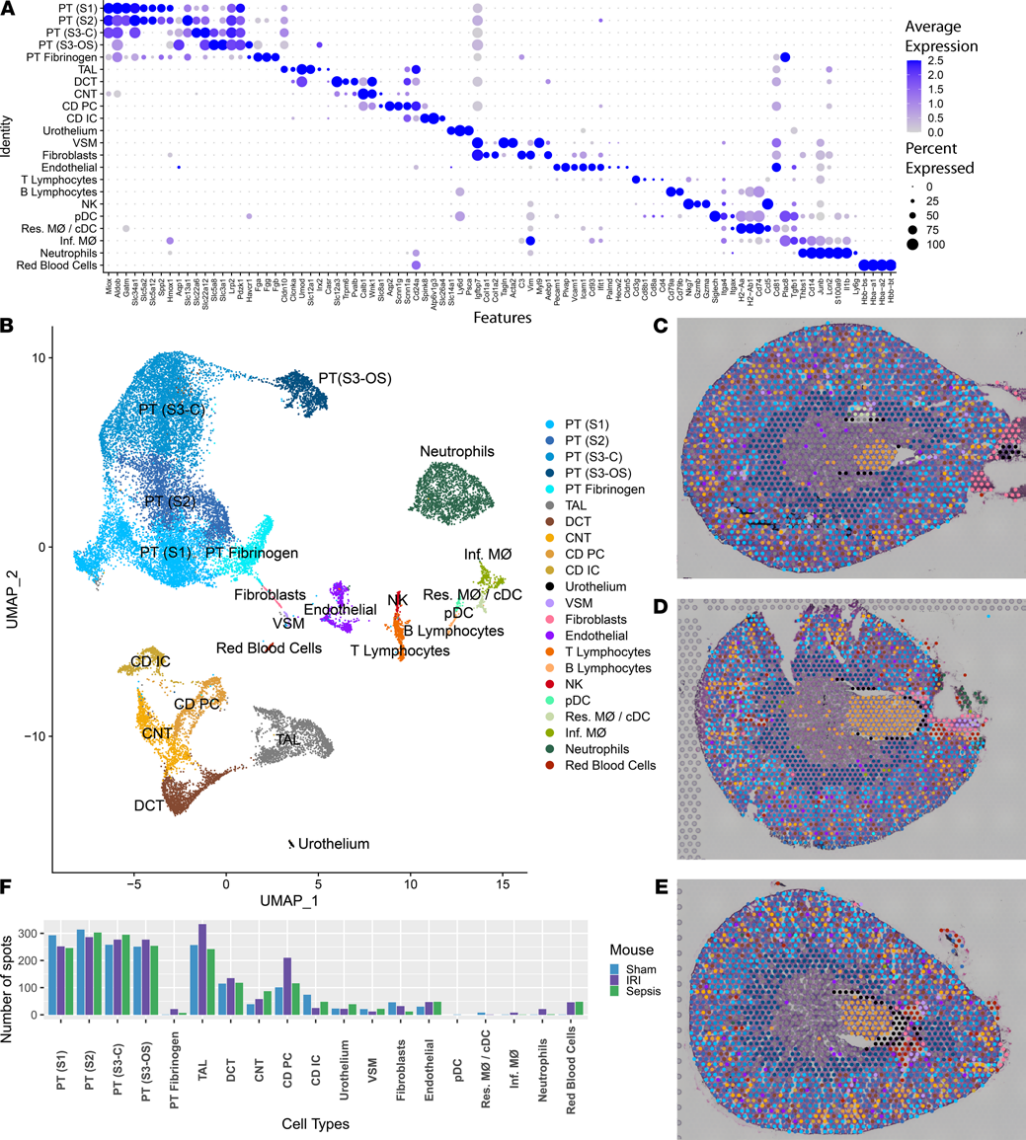
Single-cell murine data and cluster transfer to spatial transcriptomic samples. (**A**) Expression levels of markers used to define clusters in the single-cell data. The single-cell RNA sequencing data set consists of 4 murine samples: an ischemia/reperfusion injury (IRI) mouse with corresponding sham and an LPS endotoxin–administered mouse with its corresponding sham. (**B**) A UMAP displays the clusters obtained from the single-cell data. (**C**–**E**) Mapping of the single-cell clusters over the 3 murine spatial transcriptomic sections: sham, IRI, and cecal ligation puncture, respectively. (**F**) Quantitation of the number of spots mapped to each of the single-cell clusters. Raw spot counts are provided without further calculation. PT, proximal tubule; S1, S2, S3, segments of PT; S3-C, cortical section of S3; S3-OS, outer stripe section of S3; TAL, thick ascending limb; DCT, distal convoluted tubule; CNT, connecting tubule; CD, collecting duct; PC, principal cells; IC, intercalated cells; VSM, vascular smooth muscle; pDC, plasmacytoid DCs; cDC, conventional DCs; Res. MΦ, resident macrophages; Inf. MΦ, infiltrating macrophages. Each spot is 55 μm in diameter.

To improve resolution and cell-type specificity, murine scRNA-Seq data sets were clustered to build a common reference murine kidney cell atlas across models. To define cell types relevant to each model, kidneys from a sham and IRI mouse were disaggregated for scRNA-Seq. To obtain signatures of cell types relevant to the CLP model, publicly available scRNA-Seq data from an endotoxin LPS model (mouse euthanized 4 hours after endotoxin) and a corresponding sham animal were utilized (21). Although the models for the scRNA-Seq and spatial transcriptomic data sets were not identical, the scRNA-Seq data serve a broader purpose by allowing the identification of cell-type signatures within the spatial transcriptomic samples. Expected epithelial, infiltrating immune, and other cell types were identified based on their expression signature (Figure 6A and Supplemental Table 6). The common scRNA-Seq clusters from the composite UMAP of all 4 animals (IRI and sham, LPS and sham) were then mapped upon the histological section of each model (Figure 6, B–E). Beyond the traditional epithelial cell types, we identified 2 clusters of injured PT cells highly expressing either *Havcr1* or fibrinogen (*Fga, Fgb, Fgg*; ref. 22). The cluster expressing *Havcr1*, PT (S3-OS), expressed markers of the S3 PT, suggesting injury to the outer stripe region of the medulla. The PT fibrinogen cluster also had some expression of *Havcr1*, but was rich in *Fga*, *Fgb*, and *Fgg* and markers of the more cortical S1 and S2 PT markers. Both a cortical PT S3 cluster (PT-S3-C) and a medullary PT S3 cluster (PT-S3-OS) were identified in the scRNA-Seq data set (21). The DEGs between these 2 clusters were compared (Supplemental Figure 1). Although a similar number of spatial transcriptomic spots mapped to each cluster, the density of PT-S3-OS was much greater in the outer stripe than the PT-S3-C in the cortex. The PT-S3-C appeared to reside near medullary rays, although the contribution of PT S2 to the PT-S3-C cluster cannot be excluded.

We examined differential expression between the IRI and CLP models in a subset of epithelial cell clusters in the spatial transcriptomic data set (Supplemental Figure 3) and between the IRI and LPS scRNA-Seq data sets (Supplemental Figure 4). A high degree of overlap was found in each technology’s epithelial cell DEGs but not immune cell DEGs because of the paucity of spatial transcriptomic immune cell spots.

Among the immune cell clusters mapped using the scRNA-Seq data set, we identified neutrophils, NK cells, B and T lymphocytes, plasmacytoid DCs (high in *Siglech*), and 2 infiltrating macrophage clusters, *Ear2*^+^ (Ly6c^lo^) and *Chil3*^+^ (Ly6c^hi^; ref. 23). A single cluster was found to contain resident macrophages and classical DCs (high in *H2-Aa*, *H2-Ab1*), which could not be subclustered because of cell sample size. The resultant composite cluster set was used to transfer the scRNA-Seq cluster labels to the spatial transcriptomic spots.

**Figure 7:**
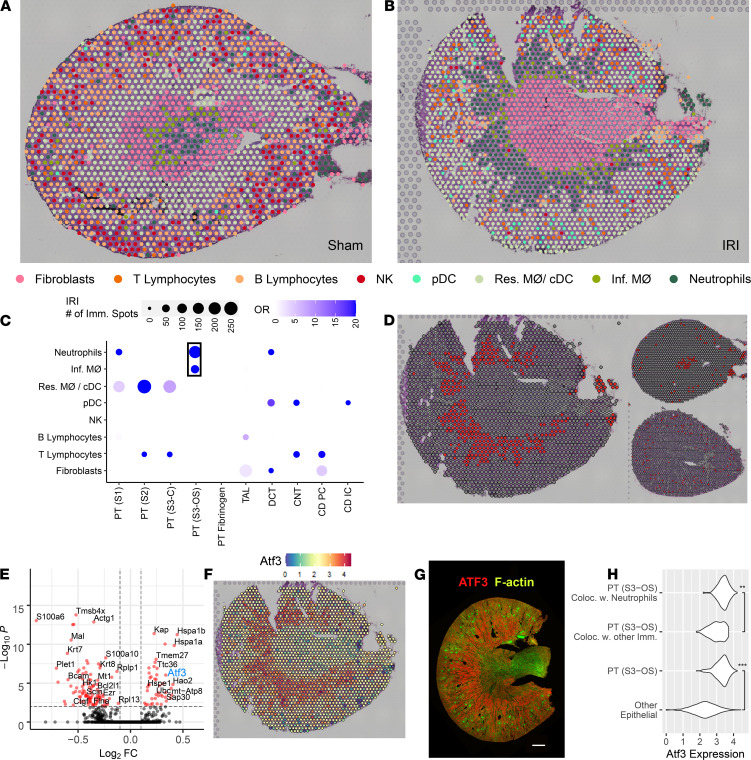
Colocalization of immune clusters in the ischemia/reperfusion injury model. (**A** and **B**) Selected single-cell immune clusters are overlaid upon the spatial transcriptomic sections for the sham and ischemia/reperfusion injury (IRI) models, respectively. Each spot was labeled with the immune cell with the highest corresponding transfer score. (**C**) The odds ratio of colocalization for each pair of immune and epithelial clusters in the IRI model when compared with the sham. Only significant comparisons are included in the dot plot as calculated by a Fisher’s exact test. Neutrophils most frequently colocalized with the PT (S3-OS) epithelial cluster. (**D**) Neutrophil plot in IRI (left), sham (top-right), and cecal ligation puncture (CLP, bottom-right). (**E**) The differentially expressed genes (DEGs) between the PT (S3-OS) spots colocalizing with neutrophils (right) and the PT (S3-OS) spots colocalizing with other immune clusters in IRI (left). (**F**) The gene expression of *Atf3* localizes to the outer stripe in IRI. (**G**) Antibody immunofluorescence of ATF3 reveals medullary outer stripe protein expression in the IRI model (*n =* 3). (**H**) The expression distribution of *Atf3* in selected clusters (**P < 10^–9^, ***P < 10^–15^) as calculated by a Fisher’s exact test. PT, proximal tubule; S1, S2, S3, segments of PT; S3-C, cortical section of S3; S3-OS, outer stripe section of S3; TAL, thick ascending limb; DCT, distal convoluted tubule; CNT, connecting tubule; CD, collecting duct; PC, principal cells; IC, intercalated cells; pDC, plasmacytoid DCs; cDC, conventional DCs; Res. MΦ, resident macrophages; Inf. MΦ, infiltrating macrophages. Each spot is 55 μm in diameter. Scale bar: 500 μm (**G**).

**Figure 8:**
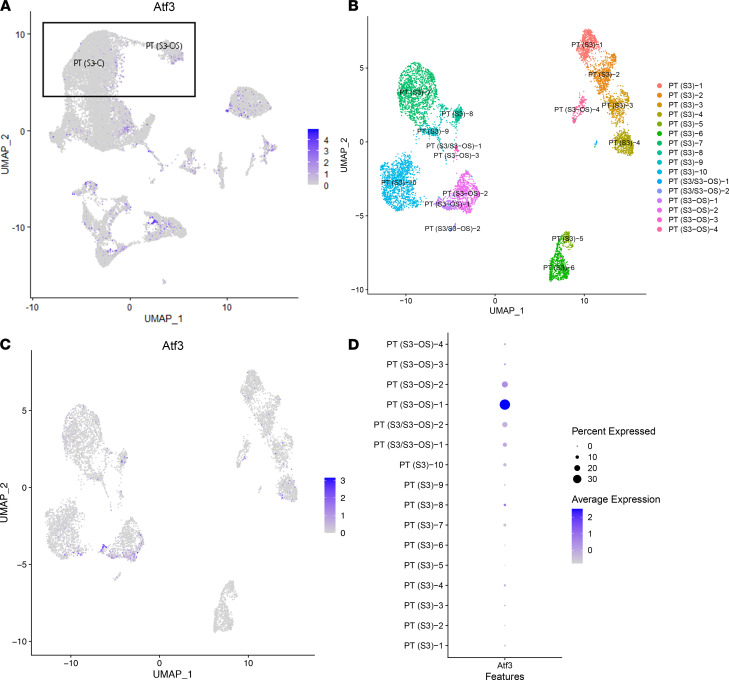
Identification of a proximal tubular single-cell subcluster expressing *Atf3*. (**A**) Feature plot of the single-cell data presenting the expression of *Atf3* with PT (S3-C) and PT (S3-OS) clusters highlighted. (**B**) A UMAP of the PT (S3-C) and PT (S3-OS) single-cell clusters, reclustered at increased resolution. (**C**) Feature plot of *Atf3* with its expression in the single-cell subclusters shows *Atf3* specifically in 1 outer stripe subcluster. (**D**) The expression of *Atf3* in all subclusters. PT, proximal tubule; S1, S2, S3, segments of PT; S3-C, cortical section of S3; S3-OS, outer stripe section of S3.

**Figure 9:**
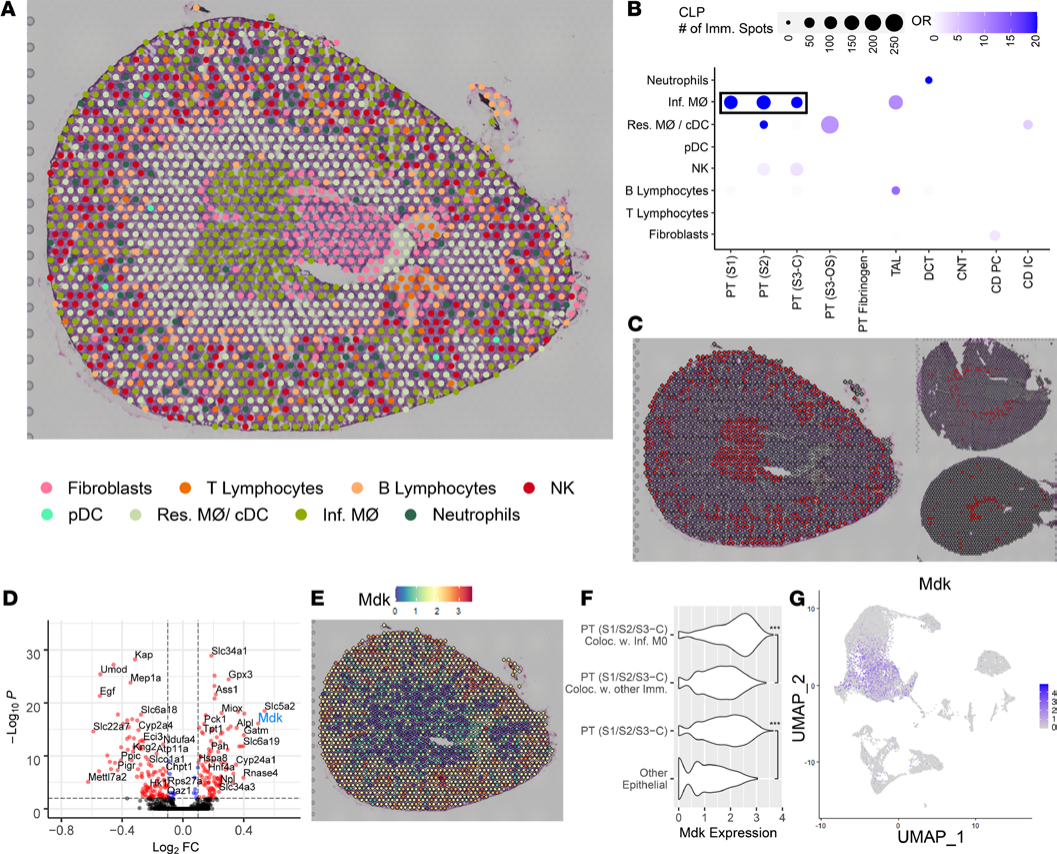
Colocalization of immune clusters in the cecal ligation puncture model. (**A**) Selected single-cell immune clusters were transferred over the cecal ligation puncture (CLP) spatial transcriptomic section. (**B**) The odds ratio of colocalization for each pair of immune and epithelial clusters in CLP when compared with the sham. (**C**) Infiltrating macrophages localized to the outer cortex and inner medulla. Infiltrating macrophage localization is depicted in the CLP (left), sham (top-right), and ischemia/reperfusion injury (bottom-right). (**D**) The differentially expressed genes (DEGs) between the PT (S1/S2/S3-C) spots colocalizing with infiltrating macrophages (right) and the ones colocalizing with other immune clusters in CLP. (**E**) The gene expression of *MdK* in CLP. (**F**) The expression distribution of *Mdk* in selected clusters (***P < 10^–15^) as calculated by a Fisher’s exact test. (**G**) The expression of *Mdk* in the single-cell data. PT, proximal tubule; S1, S2, S3, segments of PT; S3-C, cortical section of S3; S3-OS, outer stripe section of S3; TAL, thick ascending limb; DCT, distal convoluted tubule; CNT, connecting tubule; CD, collecting duct; PC, principal cells; IC, intercalated cells; pDC, plasmacytoid DCs; cDC, conventional DCs; Res. MΦ, resident macrophages; Inf. MΦ, infiltrating macrophages. Each spot is 55 μm in diameter.

The number of spots assigned to each cell type is provided in Figure 6F. Although the single-cell data set yielded an abundant immune cell population, very few spatial transcriptomic spots were assigned to those cell types. This apparent discord may be related to the dominant transcriptomic signature originating from the more abundant epithelial cells. To understand the immune cell distribution, we first applied a SPOTlight neural network analysis to each model (Supplemental Figure 5). The resulting cell distribution qualitatively agreed with the results of Figure 7. However, immune cell populations were not sufficiently represented in the neural network analysis. Thus, a transfer score system was adapted to understand the rank and relative contribution of each scRNA-Seq cell type to the signature of a given spatial transcriptomic spot (Supplemental Table 7). After suppression of the epithelial and endothelial scRNA-Seq clusters, the immune cell and fibroblast scRNA-Seq cluster labels were remapped over the histological images (Figures 7, 8, and 9).

### Colocalization of immune and epithelial cell transcriptomic signatures in spots of the IRI model.

Figure 7, A and B presents the spatial distribution of immune cell and fibroblast clusters mapped onto the sham and IRI models, respectively. A transfer score was quantitated for each scRNA-Seq cluster signature for every spatial transcriptomic spot. Only the immune cell with the highest ranked transfer score is displayed. Fibroblasts were included in the analysis as a control, i.e., the immune cell transfer score had to at least exceed that of a fibroblast to be mapped to the histological section. These new immune cell spot identities were then compared with the original epithelial cell type spot identities mapped in Figure 6.

To evaluate the colocalization of immune and epithelial cells, an odds ratio of colocalization was calculated across all spatial transcriptomic spots (Figure 8C and Supplemental Table 8) as compared with the sham mouse. The strongest association was found between the PT S3 spots of the outer stripe (PT [S3-OS]) with the neutrophil and infiltrating macrophage signature. The remaining significant cluster pairs possessed lower odds ratios or were indicative of resident macrophage colocalization. We elected to explore the relationship between the PT (S3-OS) and neutrophil migration.

The neutrophil scRNA-Seq cluster mapped specifically to spatial transcriptomic spots of the outer stripe of the medulla in the IRI model (Figure 7D). Such coclustering was not apparent in the sham and CLP models. In the IRI model, we then queried DEGs between the PT (S3-OS) spots that colocalized with neutrophils and the PT (S3-OS) spots that colocalized with any other immune cell type (Figure 7E and Supplemental Table 9). Among the DEGs was *Atf3* (activating transcription factor 3), a regulator of neutrophil migration (24). The expression of *Atf3* specifically colocalized to the outer stripe of the medulla (Figure 7F). *Atf3* protein expression similarly localized to the outer stripe (Figure 7G and Supplemental Figure 6). The expression of *Atf3* was higher in the PT (S3-OS) that colocalized with neutrophils versus the PT (S3-OS) that did not colocalize; expression of *Atf3* was also higher in the PT (S3-OS) than all other epithelial cell types (Figure 7H).

Given that a subpopulation of PT S3 expressing *Atf3* was detected, the expression of *Atf3* was assessed in the single-cell data (Figure 8A). The presence of a small, tightly grouped set of cells with high expression of *Atf3* suggested the possibility of a meaningful subpopulation. We then reclustered the cortical and outer-stripe clusters of PT S3 to better identify the subpopulation equivalent to the one detected in spatial transcriptomics. Figure 8B shows the UMAP of the subclusters; Figure 8C shows the expression of *Atf3* in those cells. Finally, Figure 8D shows we were able to identify a subpopulation of cells originating from the outer stripe that highly expressed *Atf3*. Thus, the spatial transcriptomic data facilitated the discovery of a potentially new cell type within the scRNA-Seq data set: a PT S3 cell that is signaling neutrophils.

As a control, we identified *S100a6* as a significantly downregulated gene in the PT (S3-OS) cluster that colocalized with neutrophils (Supplemental Figure 7). This gene was not highly expressed in the outer stripe by spatial transcriptomics. In the scRNA-Seq data set, *S100a6* was expressed across most immune cell types, including neutrophils.

### Colocalization of immune and epithelial cell transcriptomic signatures in spots of the CLP model.

In a corresponding analysis, we then investigated the transfer of the scRNA-Seq immune cell clusters over the CLP section (Figure 9A). The odds ratio of colocalization (Figure 9B and Supplemental Table 8) indicates macrophage signature colocalization throughout the cortical segments of PTs. Chil3^+^ and Ear2^+^ infiltrating macrophage clusters were merged to increase detection power. The spots containing a highest-ranking macrophage transfer score (i.e., strongest macrophage expression signature components) are highlighted in the 3 models (Figure 9C) and suggest that macrophage infiltration was most pronounced in the cortex of the CLP mouse as compared with the sham or IRI model. DEGs were assessed between PT spots in the CLP model that colocalized with infiltrating macrophage clusters versus PT spots that colocalized with any other immune cell or fibroblasts (Figure 9D and Supplemental Table 9). Among the top DEGs associated with macrophage colocalization was Midkine (*Mdk*), a gene encoding a growth factor associated with macrophage recruitment (25), which was more highly expressed in the outer cortex of the CLP section (Figure 9E). The expression of *Mdk* was higher in cortical PT spots that colocalized with infiltrating macrophages compared with cortical PT spots that did not colocalize with macrophages and compared with all other epithelial cell types (Figure 9F). In contrast to *Atf3*, the expression of *Mdk* in the scRNA-Seq data set (Figure 9G) did not indicate a clear subpopulation of *Mdk*-expressing PT cells, but was instead diffusely expressed across the PT. In addition to the colocalization of the macrophage signature with the PT signature, a smaller but still significant association was observed between the NK cell signature and the proximal tubular signature in the CLP model.

### Visualization of immune cells by multiplexed immunofluorescence imaging of the murine injury models.

**Figure 10:**
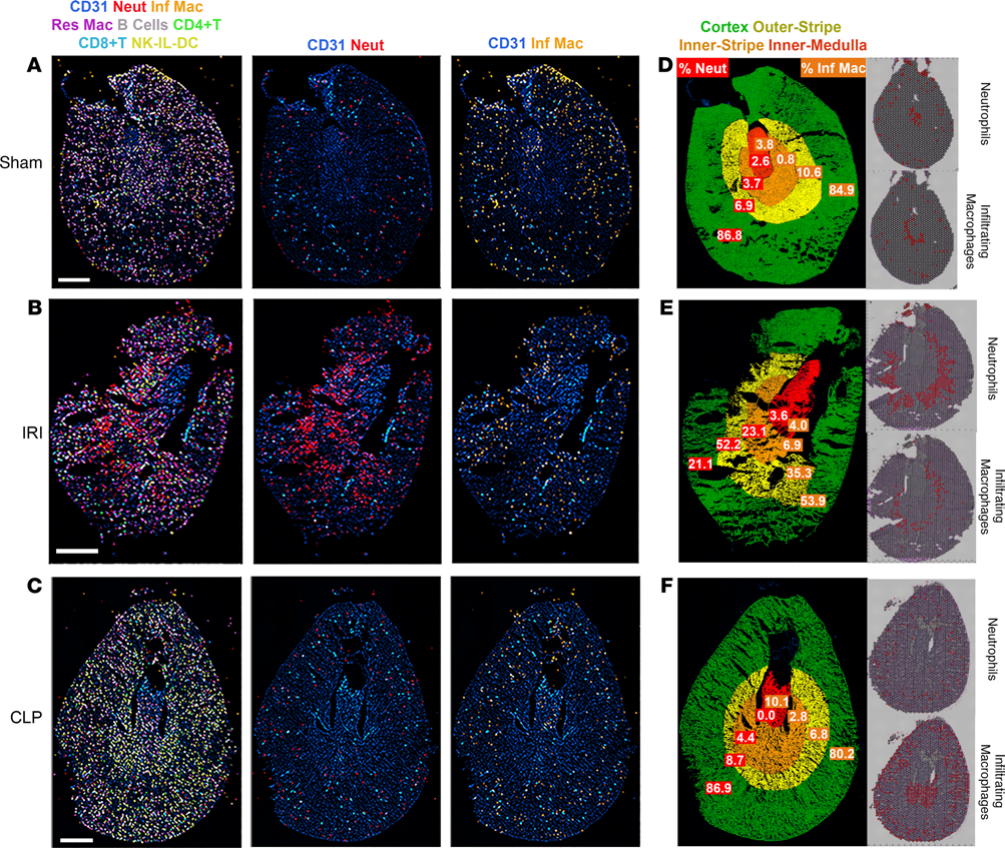
Multiplexed imaging of proteins in toto with CODEX validates the localization of immune cell clusters inferred by spatial transcriptomics. CODEX imaging for kidney sections from sham, ischemia/reperfusion injury (IRI), and cecal ligation puncture (CLP) are shown in **A**, **B**, and **C**, respectively. In the left column for all sections, spatial mapping of various immune cells (neutrophils, infiltrating and resident macrophages, B cells, CD4^+^ and CD8^+^ T cells, NK and IL cells, and DCs) are displayed using colored overlays, and CD31 staining is included for context. The definition of each cell type based on the presence and absence of markers is detailed in Supplemental Figure 8. The second column shows only CD31 and neutrophils (Neut, red), and the third column displays infiltrating macrophages (Inf Mac, orange). (**D**–**F**) show the distribution of neutrophils and infiltrating macrophages in specific regions of the kidney for sham, IRI, and CLP. The cortex, outer stripe of the medulla, inner medulla, and papilla regions were identified based on structural landmarks and annotated using region of interest (ROI) tool in ImageJ. The corresponding spatial transcriptomic signature for neutrophils and infiltrating macrophages is shown on the right side for each specimen.

To validate the localization of immune cells in each model, we performed CODEX multiplexed immunofluorescence imaging on sections from the same animals (26). Figure 10 shows the immune cell distribution in the sham, IRI, and CLP models. Supplemental Figure 8 presents the gating strategy employed in CODEX interrogation. In comparison with the sham model, neutrophils (Ly6G^+^ and CD11b^hi^) were found to predominantly infiltrate the outer stripe of the IRI section (Figure 10D). The percentage of neutrophils and infiltrating macrophages in each approximate region of the kidney (papilla, inner medulla, outer stripe of the medulla, and cortex) was quantitated (Figure 10, D–F). In the IRI model, 52.2% of all infiltrating neutrophils localized to the outer stripe. This finding is consistent with the localization of neutrophil cluster spatial transcriptomic spots to the outer stripe (Supplemental Table 10).

In the CLP model, 80.2% of infiltrating macrophages (Ly6G^–^ and CD11b^hi^) localized to the outer cortex (Figure 10F). The infiltrating macrophage signature dominated and colocalized with PT epithelial spots in the spatial transcriptomic analysis (Figure 9C), and the distribution of these macrophages localized to the outer cortex of the kidney by immunofluorescence. However, no increase in macrophage quantity was observed in the CLP model as compared with the sham model. We speculate that the activated macrophages in the CLP model contributed to the transcriptomic signature to a greater extent than the macrophages in the sham model.

A second finding in the CLP model was the infiltration of NK cells in the cortex and outer stripe of the kidney. Cells consistent with NK cells (CD45^+^, CD11b^–^, B220^–^, CD3^–^, CD4^–^, CD8^–^) localized to these regions in the CODEX assay. Further, the NK cell infiltration aligned with spatial transcriptomic colocalization of NK cells with PT S2 and PT S3 expression signatures in the CLP model (Figure 9B). In summary, the spatial transcriptomic signatures uncovered in the IRI and CLP models were supported by the immunofluorescence data.

## Discussion

In this work, we localized the transcriptomic signature of various immune cells to spatial transcriptomic spots of known renal epithelial cells in 2 models of AKI. Signatures from immune and epithelial cells were colocalized to identify possible chemotactic molecules of neutrophils and infiltrating macrophages in the IRI and CLP models, respectively. The spatial transcriptomic analysis enhanced our understanding of the single-cell data by detecting a subpopulation of injured PT cells with *Atf3* expression, which may be responsible for neutrophil chemotaxis. To accomplish this, we first optimized a workflow for the interrogation of human and murine kidney tissue with spatial transcriptomics. The technology facilitated the identification of key renal cell types and regions, including most epithelial, endothelial, and stromal cell types. The specificity of cell-type mapping was improved by concomitant snRNA-Seq or scRNA-Seq cluster analysis. The technique appeared robust, revealing strong concordance (>97%) between the transcriptomic signature of a given spot and its underlying histopathology. Supporting data were provided that AKI did not affect all regions of the kidney uniformly. The early effects of acute tubular necrosis in the IRI model led to regional changes in the transcriptomic signature at the 6-hour time point.

Single-cell sequencing has rapidly enhanced our knowledge of the kidney’s expression signature and its cell types (27), thereby helping to uncover the pathophysiology of a variety of conditions, including early diabetic nephropathy (28), the composition of a Wilm’s tumor (29), and allograft rejection (5). Similar studies in the mouse have facilitated query of murine disease state models (30) and the response to water deprivation (31). In addition, scRNA-Seq and snRNA-Seq are foundational technologies for the generation of a kidney cell atlas and detection of novel cell types in humans (2) and mice (4, 32, 33). scRNA-Seq has also aided in our understanding of kidney organ development: studies performed in organoids have shown expression of disease markers in early glomerular cells (34) and revealed that organoid expression patterns maintain strong agreement with fetal kidney expression, including lineage differences and growth signatures (35, 36). Single-cell analysis has also aided in understanding the development of fetal kidney (37–40) and myofibroblast origin (41).

In our study, we used the snRNA-Seq and scRNA-Seq data sets to effectively expand the breadth of cell types mapped to kidney tissue by spatial transcriptomics. For example, the snRNA-Seq clustering in the human kidney allowed us to differentiate interstitium spots into dominant signatures of endothelial cells, fibroblasts, and VSMCs. In the mouse, we were able to detect multiple clusters not present in the unsupervised spatial transcriptomic data set, particularly immune cells. Because there are often up to 10,000 single nuclei or cells in a given sample, the scRNA-Seq and snRNA-Seq technologies have improved sample size to detect less-represented cell types. Furthermore, the scRNA-Seq data is cell specific, rather than representative of a 55 μm spot with multiple underlying cells.

Multiple spatial transcriptomic platforms exist (42), including Slide-seq and the 10× Genomics Visium platform (6). Spatial transcriptomics have been used to establish atlases in other organs, such as the mouse brain (6) and human heart (43). In the context of disease, the spatial heterogeneity of prostate cancer (44) and melanoma in lymph nodes (45) has been described, along with the progression of amyotrophic lateral sclerosis in mice (46). The advantages of the 10× Genomics Visium platform used here include direct visualization of the underlying histology along with a higher sensitivity for gene detection. Other in situ mRNA capturing technologies maintain a higher resolution but lower sensitivity for gene expression detection. These alternatives often require the histology image from a sequential slice (47, 48) or capture smaller tissue areas (49). Other approaches to determining the spatial distribution of mRNA, such as in situ sequencing (50) or sequential in situ hybridization (51), are expensive but important ways to visualize the transcriptomic signature with spatial resolution.

The main limitation of the 10× Genomics Visium spatial gene expression platform is its resolution with a spot size of 55 μm, which will overlay multiple cells. For instance, Slide-seq has improved cellular resolution, but has not been optimized for direct visualization on the same histopathology H&E-stained specimen (42). In this work, we chose to examine the 10× Genomics Visium platform because it had the ability to create an atlas of the kidney with direct mapping of expression onto the renal structures a pathologist would assess, thereby being more readily translatable. We overcame the limitation in resolution by deconvoluting each spot with neural network analyses and a transfer score system that ranks the contribution of each scRNA-Seq cluster cell type to each transcriptomic spot signature. The limitations of this technology are further counterbalanced by its deep signature (approximately 3,000 genes detected and 10,000 reads per spot with over 16,000 genes per cluster) and the ability to directly visualize the signature over a histological image. Thus, this technique affords more than simply identifying the proximity of localized scRNA-Seq cells; it can actually identify distinct molecular signatures (as in the case of IRI) and correlate these with histology. We leveraged the advantages of the 10× Genomics Visium platform to assess regional distribution of kidney injury expression changes in both murine models. An additional limitation is that each capture area has about 5,000 spots; therefore, even if the sample covers the capture area completely, the number of spots may not be enough to cluster and differentiate all cell types. As stated above, we were able to effectively use scRNA-Seq to expand the breadth of cell types mapped.

In this work, we also employed techniques to deconvolute and colocalize transcriptomic signatures of multiple cell types within a single spot (52). A second approach was employed to detect immune cell localization and uncover potential chemotactic signals. This second form of deconvolution has been used to classify secondary contributors to a spot’s expression signature, in a similar way to what has been done in the heart (53). The immune cell population of the kidney has been extensively studied using scRNA-Seq in healthy subjects and in the context of diabetic nephropathy and lupus nephritis (3, 54, 55). In our study, we united scRNA-Seq with spatial transcriptomics to study the immune cell distribution in known murine models of AKI. Much is known of the role of immune cells in these common models of AKI. It has been shown in IRI models that infiltration of neutrophils occurs in the outer stripe of the medulla (56, 57). Further, macrophage colocalization in the S1 segment of PTs has been found protective in sepsis models (58, 59). Thus, the localization of these immune cells in the AKI models is not the novelty; instead, it is the capture of gene signatures underlying this colocalization. We used 10× Genomics Visium to predict the spatial distribution of immune cell types in the kidney and identify potential chemotactic factors associated with particular epithelial cells. The regional distribution of these cells was validated with CODEX multiplexed immunofluorescence. By design, this work is descriptive. Future investigations are needed to understand the cause-and-effect relationship of these immune cell signals in the kidney.

### Conclusions and future directions.

In summary, we present the spatially mapped transcriptomic signature of AKI in murine models and show how this methodology can be applied to human kidney tissue. In the future, this technology may assist renal pathologists in their interpretation of kidney biopsy specimens. The ability to link upregulation of a particular injury gene to a particular nephron structure is vitally important. This might allow improved classification of human AKI. Future endeavors will seek to quantitatively define the contribution of each cell-type signature to each spot and to apply this methodology to diseased human kidney tissue.

## Methods

### Human tissue, data source, and snRNA sequencing.

Publicly available snDrop-Seq RNA-Seq data were acquired for 6 Kidney Precision Medicine Project (KPMP; https://www.kpmp.org/) samples and 9 additional samples from the Washington University Kidney Translational Research Center (Gene Expression Omnibus [GEO], GSE121862). Sequencing data of nuclei for the samples were subjected to quality control metrics as previously described (2). Using Seurat 3.1 and PAGODA2 (https://github.com/kharchenkolab/pagoda2/commit/master; commit ID, e4bff4a), nuclei were reclustered and displayed as a UMAP. A single human reference nephrectomy (female aged 59 without histological evidence of kidney disease) was acquired from the Biopsy Biobank Cohort of Indiana (60).

### Murine models.

From age-matched 8- to 10-week-old 129/SvEv mice (Taconic Biosciences), tissues were acquired from a sham mouse in which the abdomen was opened and sutured back and from mice that underwent IRI or CLP. In the IRI model (61), both renal pedicles were exposed and clamped for 22 minutes or 30 minutes through a midline incision and then released. Spatial transcriptomics, histopathology, CODEX, and immunofluorescence data were acquired from the 22-minute IRI model. scRNA-Seq data were acquired from the 30-minute IRI model. In the CLP model (59), the cecum was ligated and punctured with a 25-gauge needle. Kidneys were excised upon mouse euthanization 6 hours after each procedure and frozen in OCT compound. The presence or absence of AKI was assessed on the H&E histology image and in a consecutive periodic acid–Schiff-stained section. As expected, blood urea nitrogen and creatinine measurements were not elevated in either the IRI and CLP models at the 6-hour time point.

### Slide preparation and imaging.

Slide preparation (CG000240_Demonstrated_Protocol_VisiumSpatialProtocols_TissuePreparationGuide_Rev_A, 10× Genomics) and imaging were conducted according to Visium spatial gene expression protocols (CG000241_VisiumImagingGuidelinesTN_Rev_A, 10× Genomics). Frozen transverse 10 μm sections from the human nephrectomy or each murine model were placed within the etched frames of the capture areas on the active surface of the Visium spatial slide. Tissue sections were fixed in methanol and stained with H&E. Bright-field images of stained sections in the fiducial frames were collected as mosaics of 10× fields using a Keyence BZ-X810 microscope equipped with a Nikon 10× CFI Plan Fluor objective.

### mRNA extraction and sequencing.

mRNA extraction, library preparation, and sequencing were conducted according to the Visium spatial protocols. Stained tissue sections were permeabilized for 12 minutes and mRNA was released to bind oligonucleotides on the capture areas followed by reverse transcription, second strand synthesis, denaturation, cDNA amplification, and SPRIselect cDNA cleanup (CG000239_VisiumSpatialGeneExpression_UserGuide_RevD, 10× Genomics), and then the cDNA libraries were prepared and sequenced on an Illumina NovaSeq 6000 with 28 bp + 120 bp paired-end sequencing mode.

### Murine single-cell isolation, library preparation, and sequencing.

Sham and IRI murine kidneys were transported on ice, minced, and dissociated using the Multi-Tissue Dissociation Kit 2 and a dissociator tube rotator (GentleMACS, Miltenyi Biotec). Samples were prepared according to protocol with modifications: 10 mL of RPMI 1640 (Corning) and 10% BSA (Sigma-Aldrich) were added to the mixture, then filtered, centrifuged at 300*g* for 5 minutes, and the cell pellet was resuspended in 1 mL of lysis buffer (Sigma-Aldrich). Annexin V dead cell removal was performed using magnetic bead separation after washing. The pellet was resuspended in RPMI 1640 and BSA 0.04%. A final concentration of 1 million cells/mL with over 80% viability was achieved. In a single-cell master mix with lysis buffer and reverse transcription reagents, the sample was processed according to the Chromium Single Cell 3′ Reagent Kits V3 protocol (CG000183_ChromiumSingleCell3_v3_UG_Rev-A, 10× Genomics). cDNA was synthesized and libraries were prepared. Sequencing was performed on the Illumina NovaSeq6000 with 28 bp + 91 bp paired-end mode.

scRNA-Seq was not performed for CLP mice. Instead, publicly available kidney scRNA-Seq data were downloaded from GEO (GSE154107) for mice exposed to LPS (5 mg/kg) or vehicle and euthanized at 4 hours as previously described (21).

### Murine single-cell data processing.

Data from 4 scRNA-Seq experiments (2 sham, 1 IRI, 1 LPS) were processed with Cell Ranger 2.1.0 and Seurat 3.1 in R (62). Raw base call files were demultiplexed from FASTQ files and aligned to the mm10 murine genome using STAR (63). Cells were removed based on a mitochondrial content higher than 50%, less than 200 unique genes, and the lower 10% and top 5% percentiles of unique genes distribution on each mouse. Each mouse data set was independently scaled and normalized and then merged to create a single-cell reference. After defining the clusters with a resolution of 0.65, the immune, interstitium, and endothelial clusters were reclustered with a higher resolution of 1.4.

### Spatial transcriptomics expression analysis.

Mapping and counting were performed using Space Ranger 1.0.0 with the reference genome GRCh38 3.0.0 or mmu10 provided by 10× Genomics. Space Ranger aligns the barcodes in each read with a 55 μm spot coordinate relative to the fiducial frame, associating read counts with the image. After generating counts, Space Ranger clusters spots using a graph-based clustering algorithm where a nearest-neighbor network is built in a principal components space and a Louvain modularity optimization algorithm selects the modules of highly connected spots. Finally, Space Ranger calculates differential expression between the clusters to identify DEGs using the sSeq method with a *t* test for spots with low counts and edgeR method with asymptotic β test for cells with high counts. The feature plots present expression levels that were normalized in Space Ranger. After mapping of the 3 mouse samples by Space Ranger, the data were processed in Seurat 3.1. The data were normalized by SCTransform and merged to build a unified UMAP. Glomerular spots in the sham model were manually annotated based on the composite reference UMAP. Dot plots show only above-average expression.

Seurat was used to transfer cluster labels from snRNA-Seq and scRNA-Seq to spatial transcriptomic spots in humans and mice, respectively. The integrated murine scRNA-Seq clusters were transferred to each murine spatial transcriptomic sample. The transfer procedure generates a probability score for each spot and its association with a given scRNA-Seq or snRNA-Seq cluster. The spot is assigned to the cluster with the highest score and mapped back to the spatial transcriptomic sample image. For deconvolution analyses, either a SPOTlight neural network analysis was used (52) or the dominant nonimmune clusters were suppressed in the analyses and the immune cell types with the highest probability transfer score were mapped on each mouse.

### Correlation with histology.

In human nephrectomy, 5 fields were selected at random, each composed of 237 to 245 spots, representing 40% of all spots mapped to the nephrectomy tissue. The histology underlying each spot was assessed and the percentage of concordance was determined. Spots were only counted if completely in frame. A spot was considered in alignment if any portion overlapped histology consistent with cluster identity.

### Multiplexed CODEX immunofluorescence.

Murine tissue sections of 10 μm were acquired from the same OCT block as the spatial transcriptomic sections and were collected on coverslips coated in poly-L-lysine. Sections were prepared following the protocol provided by the manufacturer, Akoya Biosciences, which is also described in detail by Goltsev et al. (26). Tissue retrieval was conducted with a 3-step hydration process. After hydration, tissues were fixed for staining. An antibody cocktail was made using the antibodies listed below and dispensed among the 3 tissues. After staining, another fixation step was performed to ensure adherence of the tissue to the slide in preparation for washing cycles during imaging. Sections were imaged at 20× resolution using the fully automated CODEX system (Akoya Biosciences) and a Keyence BZ-X810 slide scanning microscope. Samples were processed, analyzed, and visualized in CODEX-MAV software, a plugin for FIJI/ImageJ (NIH). The following antibodies and their barcodes were used for staining (all from Akoya with parenthetical catalog number): CD3-BX021 (17A2) — Cy5-RX021, CD4-BX026 (RM4-5) Atto 550-RX026, CD8a-BX029 (53-6.7) — Atto 550-RX029, CD11b-BX025 (M1/70) — Alexa Fluor 488-RX025, CD31-BX002 (MEC13.3) — Atto 550-RX002, CD45-BX007 (30-F11) — Alexa Fluor 488-RX007, CD45R/B220-BX010 (Ra3-6B2) — Alexa Fluor 488-RX010, CD169-BX015 (3D6.112) — Cy5-RX015, Ki67-BX047 (B56) — Atto 550-RX047, Ly6G-BX024 (1A8)- Cy5-RX024, MHC II–BX014 (M5/114.15.2) — Atto 550-RX014. Signal specificity was validated by ensuring the disappearance of the signal in between the imaging cycles. Gating strategy was performed similar to commonly used approaches for profiling immune cells using flow cytometry (61). The total number of cells was reported, as well as percentages of total CD45^+^ leukocytes.

### Immunofluorescence.

Tissue from the WT sham and 22-minute IRI samples (*n =* 3 per group) were harvested and stored in 4% paraformaldehyde before transfer to 0.25% paraformaldehyde. Tissues were cut at 50 μm thickness on a vibratome. An ATF3 primary antibody (Thermo Fisher Scientific, BS-0519R) was incubated overnight at a 1:100 concentration and washed with PBS. A donkey anti-rabbit Alexa Fluor 555 secondary antibody (1:200 dilution, Thermo Fisher Scientific, A32794) and FITC-phalloidin (Oregon Green 488, Thermo Fisher Scientific, O7466) with DAPI (1:200 dilution, Thermo Fisher Scientific, 62248) were subsequently incubated overnight. On the following day, tissues were washed with PBS and fixed with 4% PFA for 10 minutes. Samples were mounted on glass slides with coverslips on regular mounting media and sealed with nail polish (Electron Microscopy, 72180). Large scale tile scanning and imaging were performed on the Leica SP8 microscope and images were digitally stitched using Leica LAS X software. Four random fields (400 × 400 pixels) of the medullary outer stripe were assessed in each sample and scored according to immunofluorescence intensity of ATF3; and significance was calculated by a 2-tailed Student’s *t* test.

### Data and materials availability.

Spatial transcriptomic data are archived in GEO (GSE171406). Single-cell sequencing data are archived in GEO (GSE154107 and GSE171639).

### Statistics.

The DEGs in each comparison were found with the Seurat function FindMarkers (Wilcoxon’s rank sum test). Pathway enrichment for those genes was performed with the R packages ReactomePA (64) and ClusterProfiler (65). An odds ratio was calculated to determine the likelihood of sets of scRNA-Seq clusters colocalizing in the same spatial transcriptomic spot. Fisher’s exact tests were performed to test whether the odds ratio was greater than 1 and to verify the significance of spot distributions on regions of low expression. *P* values less than 0.05 were considered significant.

### Study approval.

This study was approved by the IRB of Indiana University (1906572234). The need for informed consent was waived given the retrospective, deidentified nature of the human subject analysis. Animal experiments and protocols were approved by the Indiana University Animal Care and Use Committee.

## Author contributions

RMF, MTE, TME, PCD, KSC, ARS, SW, LC, DJ, MJF, DB, and TH were responsible for the conception, analysis, and interpretation of data. YHC, MTE, ARS, SW, CG, XX, DJ, and KWD conducted the experiments. MTE, RMF, ARS, TME, PCD, and CZ drafted and revised the manuscript. KJK, TH, TAS, and KWD provided intellectual content. All authors gave final approval of the version to be published.

## Supplementary Material

Supplemental data

Supplemental Tables 1-10

## Version 1 05/18/2021

In-Press Preview

## Version 2 06/22/2021

Electronic publication
