# Supplementary material for: Integration of spatial and single-cell transcriptomics localizes epithelial cell–immune cross-talk in kidney injury
Source: JCI Insight. 2021 Jun 22;6(12):e147703. doi: 10.1172/jci.insight.147703 (PMC8262485; doi:10.1172/jci.insight.147703)
Supplement: Supplemental data [file jciinsight-6-147703-s070.pdf]

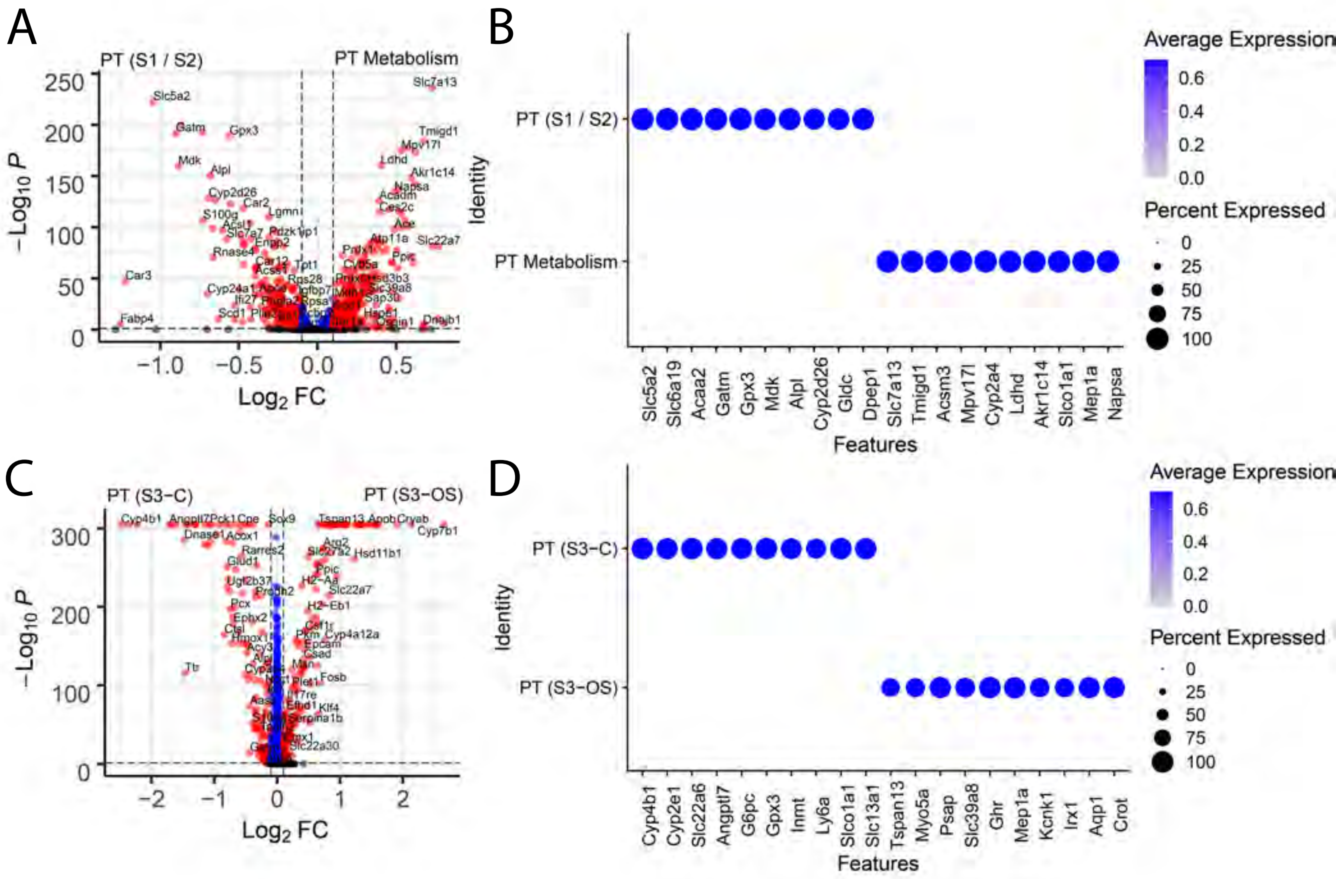

Supplemental Figure S1: Differential expression of selected unsupervised and supervised proximal tubule clusters. (A) The volcano plot reveals the differential gene expression merged across the three murine models, comparing two unsupervised clusters: PT Metabolism and PT (S1/S2). (B) The ten most significant differentially expressed genes in each cluster (PT Metabolism and PT (S1/S2)) are presented in a dot plot. (C) The volcano plot relays the differential gene expression between spatial transcriptomics mapping to two supervised clusters: PT (S3-OS) and PT (S3-C). Supervised clusters were defined by the signatures of the reference single cell dataset, but differential expression is calculated for the spots mapping to each cluster. (D) The ten most significant differentially expressed genes in each segment (PT (S3-OS) and PT (S3-C)) are presented in a dot plot. (Abbreviations: PT – Proximal Tubule; S1, S2, S3 – Segments of proximal tubule; S3-C – Cortical section of S3; S3-OS – Outer Stripe section of S3).

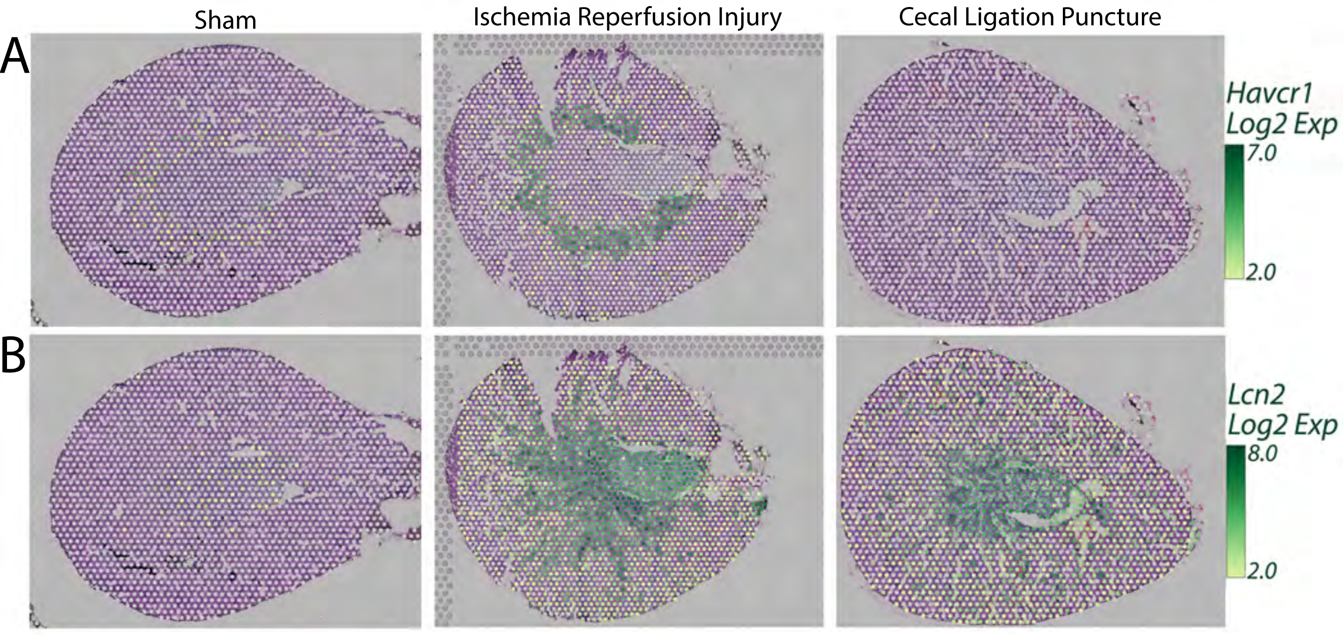

Supplemental Figure S2: Injury markers of murine acute kidney injury. (A) Spatial distribution of *Havcr1* expression overlaid upon the Sham, Ischemia Reperfusion Injury, and Cecal Ligation Puncture murine models. (B) Spatial distribution of *Lcn2* expression overlaid upon the Sham,Ischemia Reperfusion Injury, and Cecal Ligation Puncture murine models.

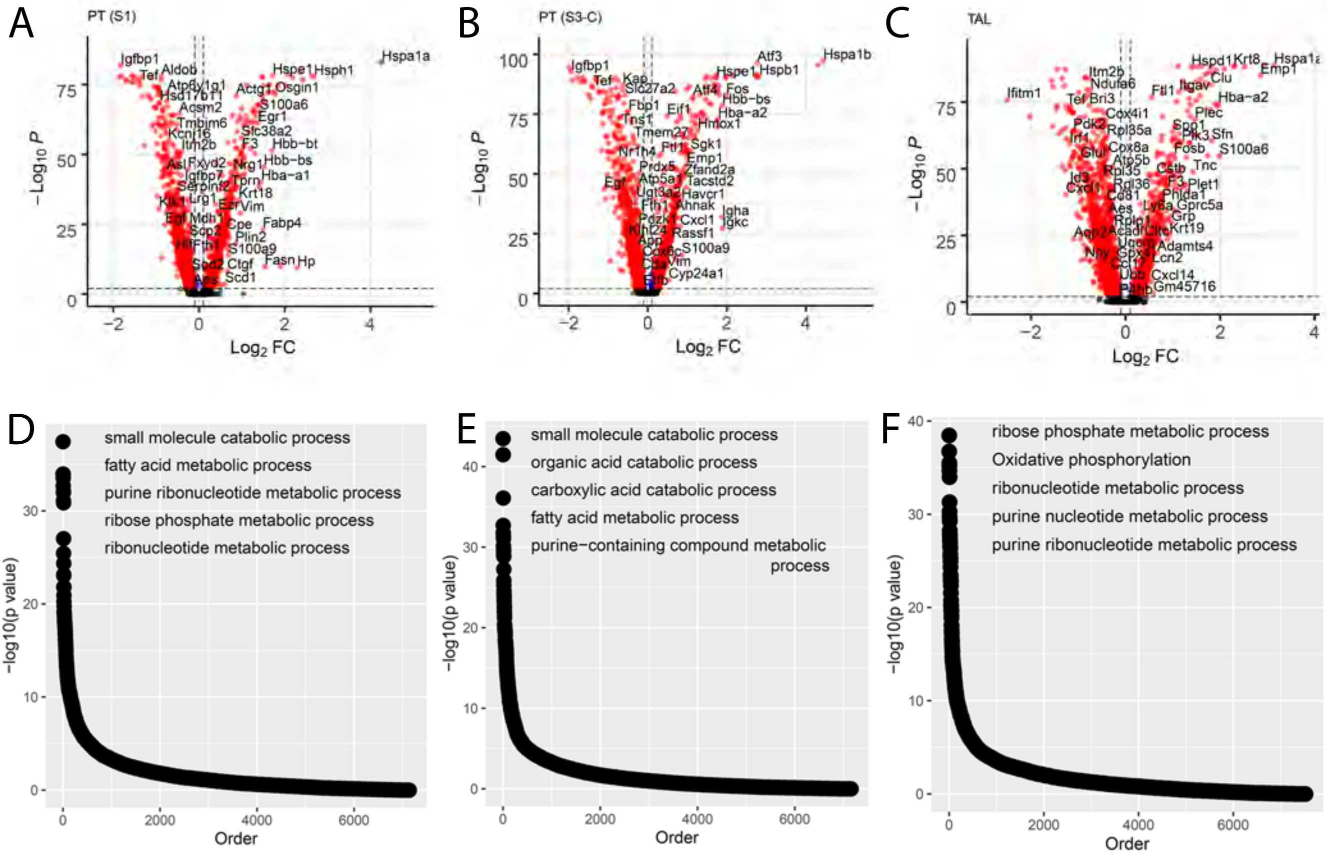

Supplemental Figure S3: Differential expression between IRI and CLP models in spatial transcriptomic spots mapped with the supervised scRNAseq cluster identities. (A-C) Differential expression between ischemia reperfusion injury and cecal ligation puncture as obtained from the spatial transcriptomic data is provided for selected clusters: PT (S1), PT (S3-C) and TAL, respectively. Genes upregulated in the IRI model are depicted on the right. Those upregulated in CLP are on the left. (D-F) Pathways enriched for the differentially expressed genes in the respective segment PT, (S1), PT (S3-C) and TAL, respectively. The top five pathways are annotated in the figure. (Abbreviations: PT – Proximal Tubule; S1– first segment of the proximal tubule; S3-C – Cortical section of S3; TAL – Thick Ascending limb.

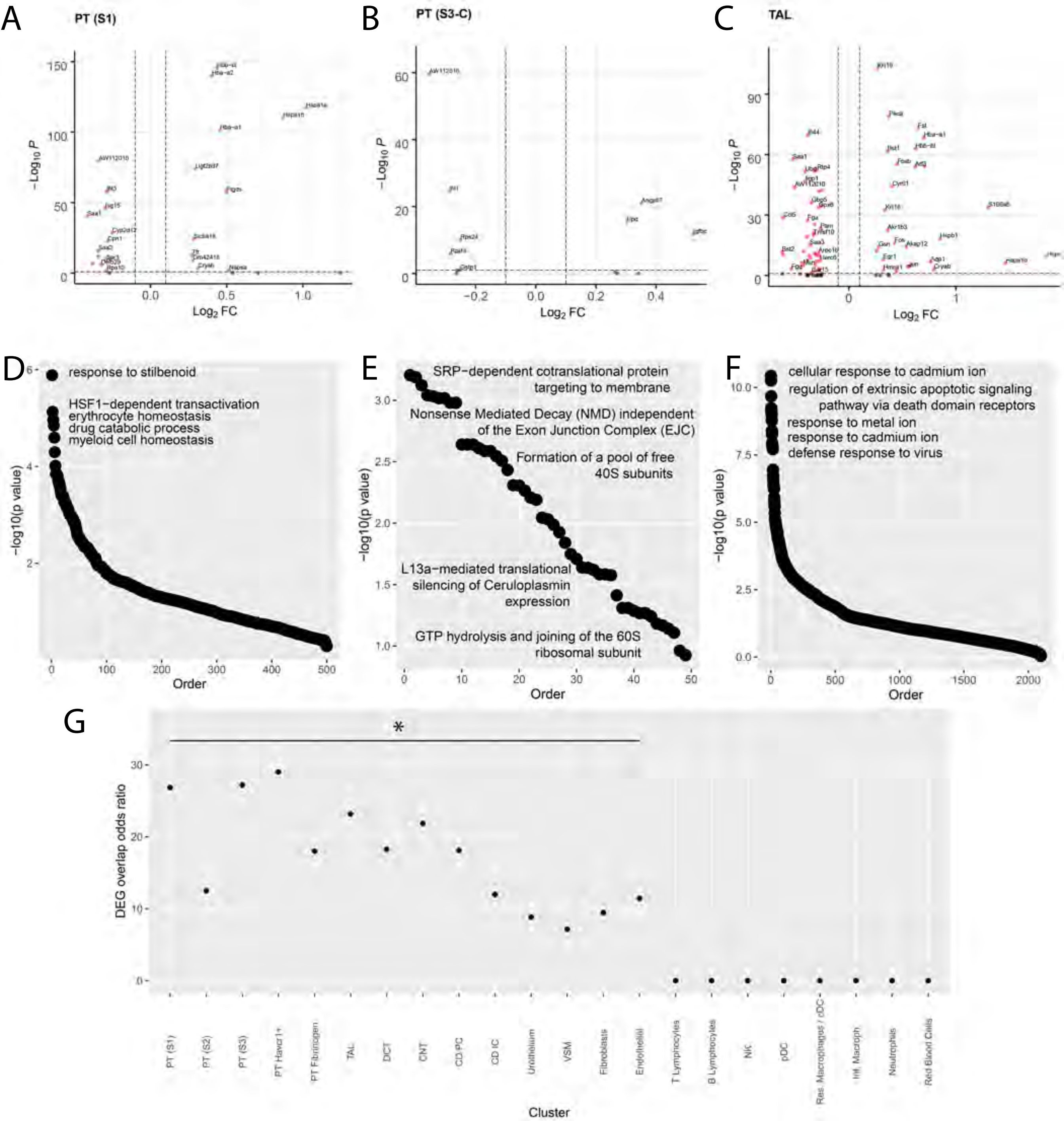

Supplemental Figure S4: scRNAseq differential expression between IRI and LPS models. (A-C) Differential expression in the single cell data between Ischemia Reperfusion Injury (IRI) and Lipopolysaccharide (LPS) murine kidneys in three clusters: PT (S1), PT (S3-C) and TAL, respectively. (D-F) Pathways enriched for the differentially expressed genes in the respective segment PT (S1), PT (S3-C) and TAL, respectively. The top five pathways are annotated in the figure. (G) Odds ratio of overlap of differentially expressed genes between single cell and Spatial Transcriptomics in all clusters. All Odds Ratio above zero are statistically significant. (Abbreviations: PT – Proximal Tubule; S1, S2, S3 – Segments of proximal tubule; S3-C – Cortical section of S3; S3-OS – Outer Stripe section of S3; TAL – Thick Ascending limb; DCT – Distal Convoluted Tubule; CNT – Connecting Tubule; CD – Collecting Duct; PC – Principal Cells; IC – Intercalated Cells; VSM – Vascular Smooth Muscle; NK – Natural Killer Cells; pDC - Plasmacytoid Dendritic Cells; cDC - Conventional Dendritic Cells; Res. MΦ – Resident Macrophages; Inf. MΦ – Infiltrating Macrophages).

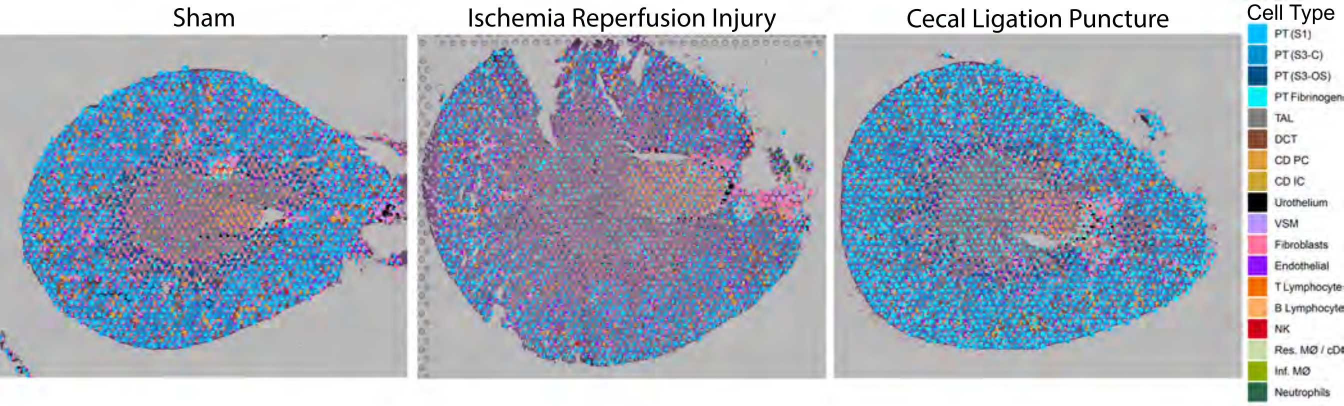

Supplemental Figure S5: SPOTlight neural network analysis of the murine models. The spatial transcriptomic signature was deconvoluted with SPOTlight for the Sham, Ischemia Reperfusion Injury, and Cecal Ligation Puncture murine models. Each pie chart represents the contribution of each cell type to the transcriptomic signature of the underlying spot. Only cell types contributing to at least 10% of the spot signature are displayed. Epithelial signatures dominate the expression patterns of each spot. Immune cell localization was minimal in this analysis. (Abbreviations: PT – Proximal Tubule; S1, S3 – Segments of proximal tubule; S3-C – Cortical section of S3; S3-OS – Outer Stripe section of S3; TAL – Thick Ascending limb; DCT – Distal Convoluted Tubule; CD – Collecting Duct; PC – Principal Cells; IC – Intercalated Cells; VSM – Vascular Smooth Muscle; NK – Natural Killer Cells; pDC - Plasmacytoid Dendritic Cells; cDC - Conventional Dendritic Cells; Res. MΦ – Resident Macrophages; Inf. MΦ – Infiltrating Macrophages). Each spot is 55 μm in diameter.

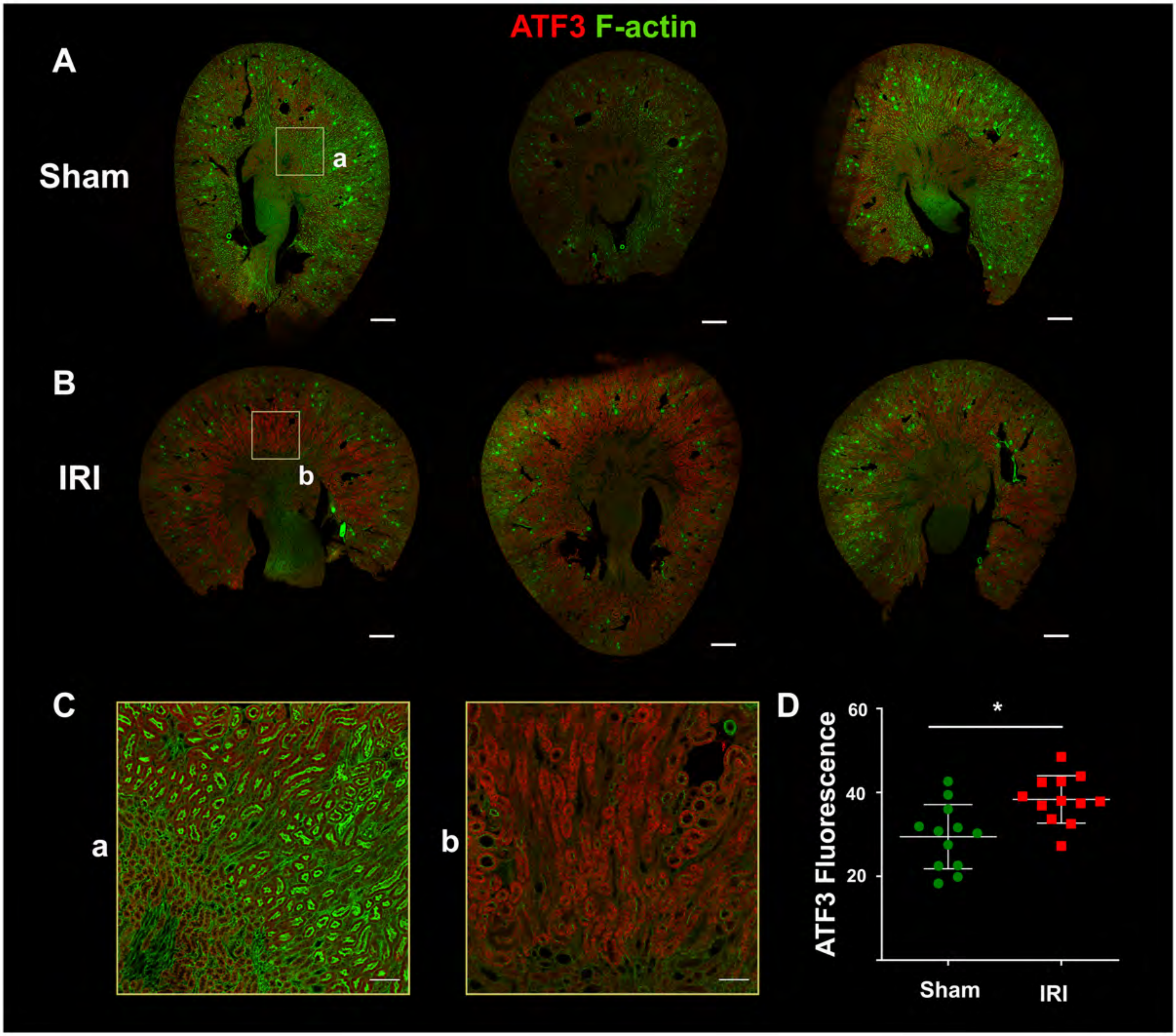

Supplemental Figure S6: Atf3 immunofluorescence in sham and IRI mice. (A-B) Large scale imaging from sham and ischemia-reperfusion injury (IRI) sections were stained for Atf3 and FITC-Phalloidin to label F-actin (N = 3 per group). Scale bar in the top two rows is 500 μm. (C) High magnification images of Atf3 immunofluorescence in the region of the outer medulla as indicated by the insets a (sham) and b (IRI). Scale bars for insets are 100 μm. (D) Quantitation of the immunofluorescence intensity in the outer stripe of the medulla. Four fields per section were measured with mean Atf3 fluorescence intensity. * = $P < 0.05$.

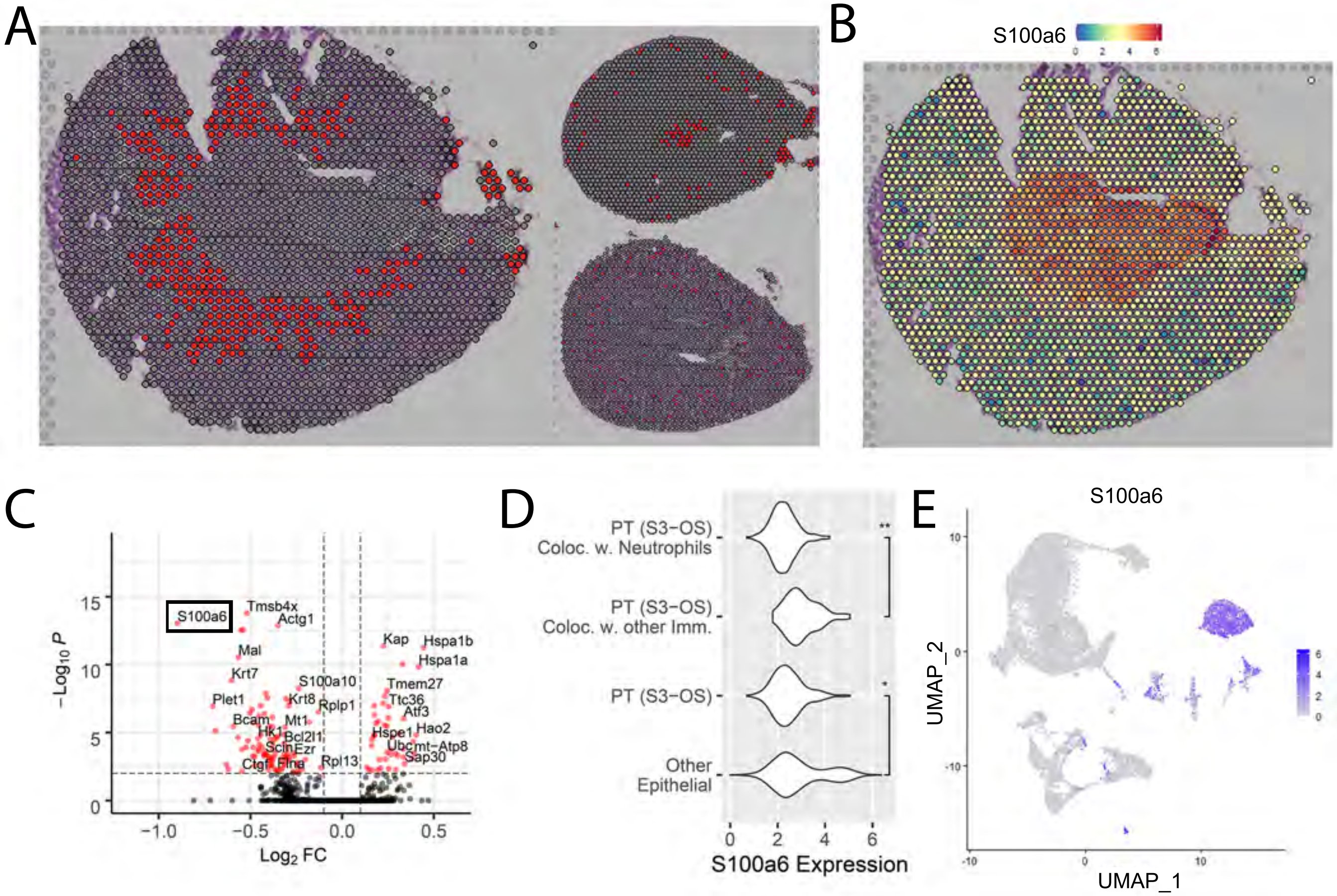

Supplemental Figure S7: Analysis of S100a6 colocalization with Neutrophils in Ischemia Reperfusion Injury. As a negative control, the expression distribution of s100a6 was assessed in ischemia-reperfusion. (A) Highlight of Neutrophils in Ischemia Reperfusion Injury (left), Sham (top-right) and Cecal Ligation Puncture (bottom-right) murine models. (B) Feature plot of the expression level of s100a6 in Ischemia Reperfusion Injury. (C) Volcano plot presenting the Differentially Expressed Genes (DEGs) between the PT (S3-OS) spots colocalizing with Neutrophils (right) and the ones colocalizing with other immune clusters in IRI (left). (D) Violin plot comparing the expression distribution of s100a6 in selected clusters (* - p<10-2, ** - p<10-9). (E) Feature plot presenting the expression of s100a6 in the single cell data. (Abbreviation: PT (S3-OS) – S3 Segment of Proximal tubule located in Outer Stripe).

A

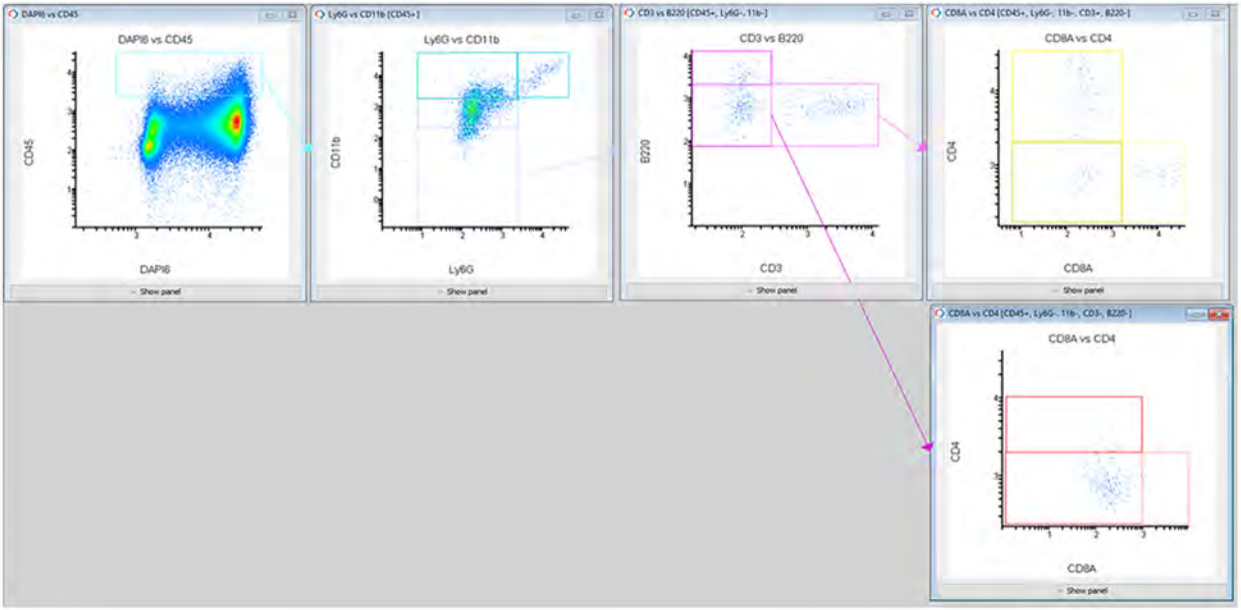

B

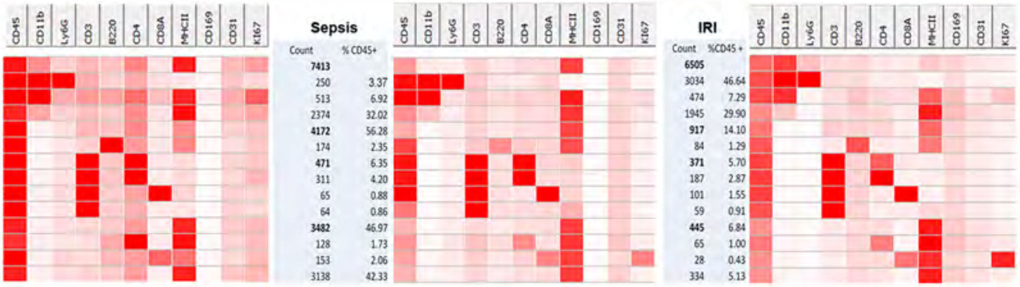

Cell Type	Sham Count	Sham % CD45+	Sepsis Count	Sepsis % CD45+	IRI Count	IRI %CD45 +
**CD45+**	**4954**		**7413**		**6505**	
**Ly6G+, CD11b hi** (Neuts)	209	4.22	250	3.37	3034	46.64
**Ly6G-, CD11b hi** (Inf Macs)	1111	22.43	513	6.92	474	7.29
**Ly6G-, CD11b lo** (Res Macs)	2964	59.83	2374	32.02	1945	29.90
**Ly6G-,CD11b-**	**541**	10.92	**4172**	56.28	**917**	14.10
**B 220+** (B Cells)	58	1.17	174	2.35	84	1.29
**CD3+** **(T cells)**	**206**	4.16	**471**	6.35	**371**	5.70
CD4+	116	2.34	311	4.20	187	2.87
CD8a+	34	0.69	65	0.88	101	1.55
**CD4-, CD8-**	44	0.89	64	0.86	59	0.91
**CD3-**	**258**	5.21	**3482**	46.97	**445**	6.84
**CD4+ (MHCII+-DCs)**	17	0.34	128	1.73	65	1.00
**CD8a+ (MHCII+-DCs)**	10	0.20	153	2.06	28	0.43
NKs, ILCs, DCs	224	4.52	3138	42.33	334	5.13

Supplemental Figure S8: Gating strategy for CODEX analysis and multiplex profile of the immune cells. (A) Representative example of the gating strategy that was utilized for CODEX analysis. This strategy is based on flow cytometry profiling of immune cells. (B) Heatmaps depicting the relative distribution of median intensity fluorescence for each of the immune cell population in the three samples. The markers used are displayed in each column on the top, and the supervised analysis of immune cell populations based on the analysis used in (A) is shown in the left column, in a hierarchal pattern that follows the gating strategy. The number of cells in each population, as well as the percent of the total CD45+ cells are also shown. Of note, MHCII was not used in the supervised analysis and its level of expression in each cell type served for validation of the analytic strategy.
